# Hidden Disease Susceptibility and Sexual Dimorphism in the Heterozygous Knockout of Cyp51 from Cholesterol Synthesis

**DOI:** 10.1371/journal.pone.0112787

**Published:** 2014-11-13

**Authors:** Monika Lewinska, Peter Juvan, Martina Perse, Jera Jeruc, Spela Kos, Gregor Lorbek, Ziga Urlep, Rok Keber, Simon Horvat, Damjana Rozman

**Affiliations:** 1 Center for Functional Genomics and Bio-Chips, Faculty of Medicine, University of Ljubljana, SI-1000, Ljubljana, Slovenia; 2 Medical Experimental Centre, Institute of Pathology, Faculty of Medicine, University of Ljubljana, SI-1000, Ljubljana, Slovenia; 3 Institute of Oncology Ljubljana, Zaloška cesta 2, SI–1000, Ljubljana, Slovenia; 4 Department of Animal Science, Biotechnical Faculty, University of Ljubljana, Groblje 3, 1230, Domžale, Slovenia; 5 National Institute of Chemistry, Hajdrihova 19, 1000, Ljubljana, Slovenia; Clermont Université, France

## Abstract

We examined the genotype-phenotype interactions of *Cyp51^+/−^* mice carrying one functional allele of lanosterol 14α-demethylase from cholesterol biosynthesis. No distinct developmental or morphological abnormalities were observed by routine visual inspection of *Cyp51^+/−^* and *Cyp51^+/+^* mice and fertility was similar. We further collected a large data-set from female and male *Cyp51^+/−^* mice and controls fed for 16 weeks with three diets and applied linear regression modeling. We used 3 predictor variables (genotype, sex, diet), and 39 response variables corresponding to the organ characteristics (7), plasma parameters (7), and hepatic gene expression (25). We observed significant differences between *Cyp51^+/−^* and wild-type mice in organ characteristics and blood lipid profile. Hepatomegaly was observed in *Cyp51^+/−^* males, together with elevated total and low-density lipoprotein cholesterol. *Cyp51^+/−^* females fed high-fat, high-cholesterol diet were leaner and had elevated plasma corticosterone compared to controls. We observed elevated hepatocyte apoptosis, mitosis and lipid infiltration in heterozygous knockouts of both sexes. The *Cyp51^+/−^* females had a modified lipid storage homeostasis protecting them from weight-gain when fed high-fat high-cholesterol diet. Malfunction of one *Cyp51* allele therefore initiates disease pathways towards cholesterol-linked liver pathologies and sex-dependent response to dietary challenge.

## Introduction

**Figure 1:**
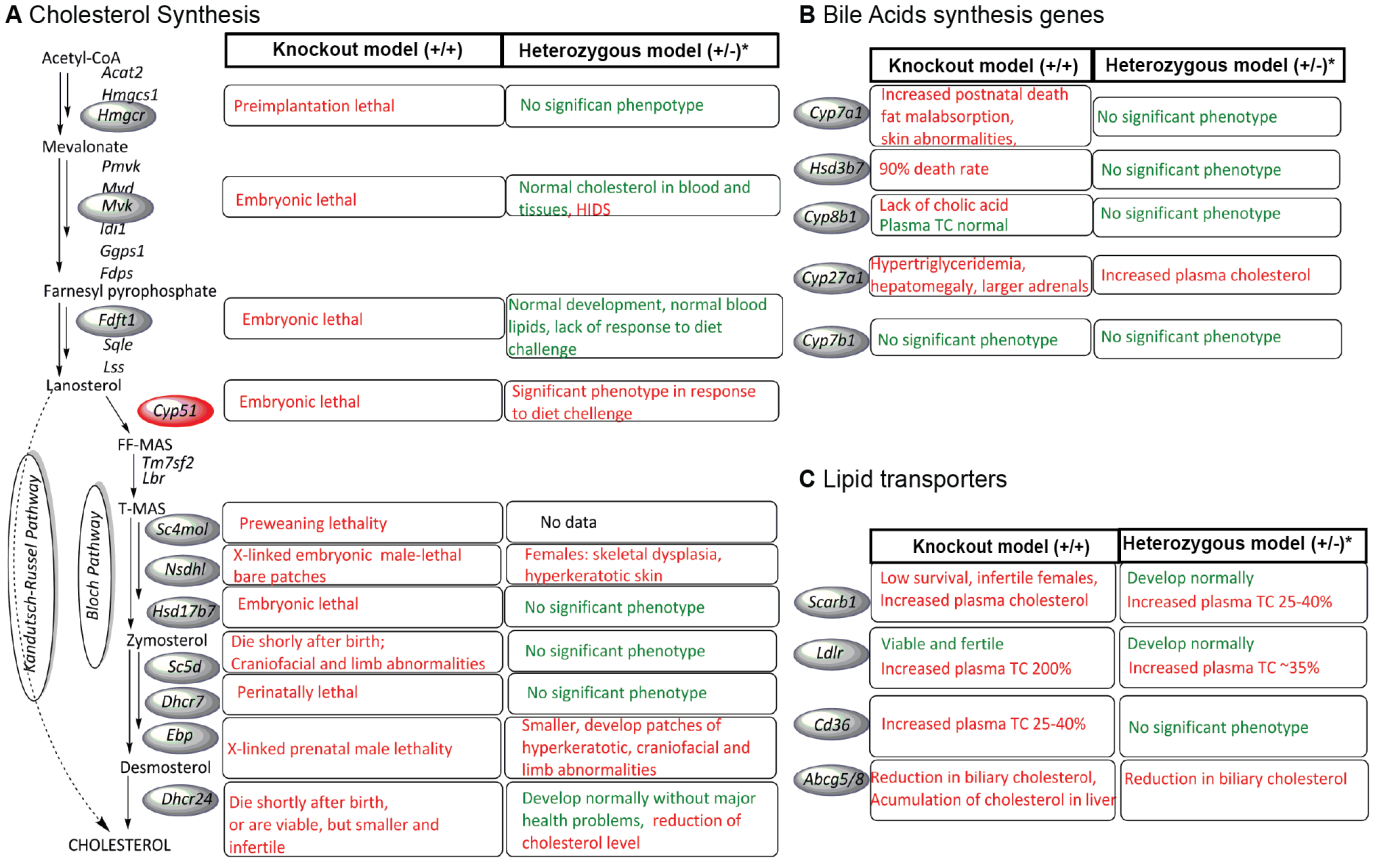
Characteristics of knock-out and heterozygous knock-out mouse models. Figure shows mouse characteristics in the cholesterol synthesis (A), bile acid synthesis pathways (B) and lipid transporters (C). In red are marked disease phenotypes, while in green are marked mild/no phenotype. *The heterozygous knock-out models often were not thoroughly investigated. Gene names and references to literature describing mouse models: *Acat2* acetyl-coenzyme A acetyltransferase 2; *Hmgcs1* 3-hydroxy-3-methylglutaryl-coa synthase 1; *Hmgcr* 3-hydroxy-3-methylglutaryl-coa reductase [55]; *Pmvk* phosphomevalonate kinase; *Mvd* diphosphomevalonate decarboxylase; *Mvk* mevalonate kinase [56]; *Idi1* isopentenyl-diphosphate delta isomerase 1; *Ggps1* geranylgeranyl diphosphate synthase 1; Fdps farnesyl diphosphate dynthase; *Fdft1* farnesyl-diphosphate farnesyltransferase 1 [57]; *Sqle* squalene epoxidase; *Lss* lanosterol synthase; *Cyp51* lanosterol 14-alpha-demethylase [17], *Tm7sf2* C-14 sterol reductase; *Lbr* lamin-B receptor; *Sc4mol* methylsterol monooxygenase 1*(WTSI); *Nsdhl* NAD(P) dependent steroid dehydrogenase-like [58], [59]; *Hsd17b7* hydroxysteroid (17-beta) dehydrogenase 7 [60]; *Sc5d* lathosterol oxidase [61]; *Dhcr7* 7-dehydrocholesterol reductase [62]; *Ebp* emopamil binding protein (sterol isomerase) [63]; *Dhcr24* 24-dehydrocholesterol reductase [41], [64]; *Cyp7a1* cholesterol 7-alpha-monooxygenase [65], [66]; *Hsd3b7* 3 beta-hydroxysteroid dehydrogenase type 7 [67]; *Cyp8b1* 7-alpha-hydroxycholest-4-en-3-one 12-alpha-hydroxylase [68]; *Cyp27a1* Sterol 26-hydroxylase [69]; *Cyp7b1* 25-hydroxycholesterol 7-alpha-hydroxylase [70]; *Scrab1* scavenger receptor class b, member 1 [71]; *Ldlr* low density lipoprotein receptor [72]; *Cd36* fatty acid translocase [73], [74]; *Abcg5* ATP-binding cassette sub-family g member 5; *Abcg8* ATP-binding cassette sub-family g member 8 [75].

Cholesterol, an essential compound of cell membranes, regulates permeability, fluidity, and membrane signaling capacity [1], is a precursor of steroid hormones and bile acids, and plays an important role in cell proliferation [2], [3]. Cholesterol originates from two sources – the dietary intake (30–50%) and *de novo* synthesis (50–70% in men) [4]. Its abnormal blood concentration leads to the increased risk of heart diseases and brain strokes. Thus, regulation on the cellular level and on the level of the entire organism is essential [5]. The lipid homeostasis is performed mainly by the liver, the major organ of lipid clearance [6] and synthesis. Almost 40% of the cholesterol is synthesized in the murine liver [7], and the pathway is well conserved in mammals. The loss of function of genes from cholesterol synthesis, metabolism or transport results in lethality or other serious conditions, where the severity of the phenotype depends on the position of gene in the pathway [8], [9], [10], [11]. Most murine studies focus on the complete knockout models that are unlikely to be found in humans, due to the lethal developmental phenotype, while mice heterozygous for the cholesterol-linked genes seldom present a distinct phenotype (Figure 1). However, the cholesterol homeostasis in humans exhibits examples where abnormalities manifest with the heterozygous variants, such as in the genes of cholesterol synthesis (*HMGCR, DHCR7, DHCR24* and *CYP51A1),* where polymorphisms associate with preterm delivery or low birth weight [12], [13], [14].

The focus of our study is lanosterol 14α-demethylase CYP51, a cytochrome P450 from the cholesterol biosynthesis pathway. In humans, the *CYP51A1* shows low nucleotide variability compared to other genes of the pathway and other related cytochrome P450 genes [15]. The mouse C*yp51* gene is 89% identical to the human counterpart [16]. The complete knockout of *Cyp51* is embryonically lethal in mice [17]. In humans homozygous *CYP51A1* dysfunctions have not been detected so far, as they probably spontaneously abort in early development. *Cyp51* is likely not essential for normal spermatogenesis [18] even if the products of lanosterol demethylation might serve as signaling sterols [19]. In humans, *CYP51A1* was hemizygously deleted in a family with cerebral cavernous malformations [20], and the gene was proposed as a candidate for the cause of pediatric cataracts [21]. The *CYP51A1* heterozygous common variant (rs6465348) associates with the decreased birth weight in preterm babies and with the changed lipid profile in pregnant women [12]. Due to the crucial role of cholesterol synthesis for organisms’ integrity, the reported associations of *CYP51A1* polymorphisms with brain function or early development likely represent only a portion of potential malformations caused by dysfunction of CYP51.

To assess the global role of decreased expression of *Cyp51* by 50%, we investigated a large colony of *Cyp51* heterozygous knockout (*Cyp51^+/−^*) mice of both sexes challenged with three diets differing in the fat and/or cholesterol content. The statistical modeling revealed novel associations between the *Cyp51* genotype, the nutrition and the lipid homeostasis. Those factors, together with histopathology pinpointed to the liver as the most prominent disease target organ.

## Materials and Methods

### Animals

Heterozygous males *Cyp51^+/−^* (B6.129SV-Cyp51<tm1.1Bfro>) were obtained from the Department of Animal Science, Biotechnical Faculty, University of Ljubljana and mated with C57BL/6JOlaHsd females (Harlan, Italy). The experiments were performed at the Medical Experimental Center, Medical Faculty, University of Ljubljana. All the animals of over 95% of C57BL/6J background were housed 3–7 per cage (825 cm^2^ floor space) on Lignocel ¾ bedding material (Germany) in a 22–23°C room, 55±10% humidity and a 12 h light/dark cycle (illumination between 07.00 am −07.00 pm) with free access to food and acidified tap water (pH = 3). The experiment was approved by the Ethical Committee for laboratory animals of the Republic of Slovenia and Veterinary Administration of the Republic of Slovenia granted permit numbers 34401-31/2011/4 and 34401-29/2011/3. The experiment was conducted in accordance with the Directive 2010/63/EU on the protection of animals used for scientific purposes, as well as in accordance with National Institutes of Health guidelines for work with laboratory animals.

### Diet regime

Litters were weaned at age of 3 weeks, genotyped as described [17] and randomly distributed to one of the diets for period of 16 weeks (8–15 mice/group; each group represents mice of specific gender and genotype on one of the diets):

- LFnC (Low Fat no Cholesterol diet) (Item no. 1324 LFnC GmbH & Co. KG, Germany), a control diet with no cholesterol, supplying 2844 kcal/kg where 65% of the calories derives from carbohydrates, 24% from proteins and 11% from fat.
- HFnC (High Fat no Cholesterol diet) – High Fat Clinton-Cybulsky rodent diet without cholesterol (D12106C, Research Diets INC, USA) supplying 4056 kcal/kg where 40.0% of the calories derives from carbohydrates, 20.0% from proteins and 39.9% from fat.
- HFC (High Fat diet with Cholesterol) - High Fat Clinton-Cybulsky rodent diet with 1.25% cholesterol (D12108C, Research Diets INC, USA) supplying 4056 kcal/kg where 40.0% of the calories derives from carbohydrates, 20.0% from proteins and 39.9% from fat.

### Organ and blood collection

A total of 142 mice were sacrificed after a 5 h fast with cervical dislocation during the light period (11.30 am −2.30 pm) to limit diurnal variability. During the autopsy the mice were weighed; organs were removed, trimmed of adipose tissue, weighed and patho-morphologically examined. For the histology, left lateral lobe (*lobus sinister lateralis*) was fixed in 4% formalin and embedded in paraffin, sectioned (5 µm) and stained with hematoxylin and eosin. The rest of the liver was cut into small slices, snap frozen in liquid nitrogen and stored at −80°C for further analyses. Immediately after cervical dislocation, the blood was taken from the right ventricle, collected into lithium-heparin coated tubes, and centrifuged for 15 minutes at 4°C at 3000×g; plasma was collected, snap frozen in liquid nitrogen, and stored at −80°C.

### Histology

Liver sections of each mouse were scored individually for different parameters such as size, shape and polymorphism of the hepatocytes, nuclei and nucleoli, granulation of the cytoplasm, presence of inflammation, cholestasis, steatosis, apoptosis, mitosis, and potential abnormalities of bile ducts. Histological scoring was performed on hematoxylin and eosin stained sections in a blinded fashion by a pathologist, with a scoring system for steatosis as follows: no steatosis was assigned when less than 5% of hepatocytes contained lipid droplets, mild steatosis when 5–25% of hepatocytes contained lipid droplets, moderate when 25–50% of hepatocytes contained lipid droplets, severe steatosis when lipid droplets were present in 50–75% of hepatocytes, and very severe when lipid droplets were present in more than 75% of hepatocytes. Apoptoses and mitoses were counted on the whole liver section. Liver samples were taken in the same way for every mouse (vertical cut in the middle of the right lobe) to exclude variability due to technical preparation of the sample. Apoptotic cells were recognized by characteristic morphological features, such as chromatin condensation, cell shrinkage, and nuclear fragmentation [22]. Mitotic figures were identified according to the criteria proposed by van *Diest et al* [23]. Only cells with clear morphological features of metaphase, anaphase, and telophase were counted, avoiding apoptotic and hyperchromatic nuclei.

### Blood parameters

Plasma was analyzed by the Veterina Le Marechal d.o.o, Ljubljana by Architect Ci 8200 (Abbott Corporation, USA) using methods recommended by The International Federation of Clinical Chemistry and Laboratory Medicine (IFCC) and the Clinical and Laboratory Standards Institute (CLSI), with original or recommended reagents: Total Cholesterol (TC) with Architect/Aeroseth 7D62 (Abbott), High Density Lipoprotein Cholesterol (HDL) with Architect/Aeroseth 3K33-20 (Abbott), Triglycerides (TG) with Architect/Aeroseth 7D74 (Abbott) and Free Fatty Acids (FFA)/Non-Esterified Fatty Acids NEFA-HR (2) 436–91995 (Wako Chemicals GmbH). Non-HDL cholesterol (LDL) was calculated using the Friedewald equation [24]: *LDL* [mmol] = TC [mmol]-HDL [mmol]-(TG [mmol])/2.2).

Corticosterone was measured in plasma (5 animals/group, in duplicates) by a Corticosterone (Rat/Mouse) ELISA kit (EIA-5186, DRG Instruments GmbH) according to the manufacturer’s instructions. Testosterone measurements in the males’ plasma were measured with the Testosterone (Rat/Mouse) ELISA kit (EIA-5179, DRG Instruments GmbH) according to the manufacturer’s instructions. The average absorbance of samples was used for curve-fitting with Four Parameter Logistics using the ReaderFit software (https://www.readerfit.com/).

### Gene expression analyses

The total RNA was extracted from 30 mg of liver using the QuickGene RNA tissue kit SII (FujiFilm). RNA concentration was measured with NanoDrop ND-1000 and the quality (RNA Integrity Number) checked on RNA chips (Agilent). The total RNA (3 µg) in water was treated with amplification grade DNase I (Roche) by standard protocol and used immediately for the cDNA synthesis with the SuperScript III cDNA Synthesis Kit (Invitrogen). Real-time qPCR was performed with the LightCycler 480 using SYBR Green I Master according to instructions (Roche Applied Science). Primers were designed using the Primer3 software in a way that they would span over the intron and validated with melting and standard curve analyses (Table S1). The reaction was performed in a 5 µl volume using 3.75 ng of cDNA, 1.15 µl of water, 2.5 µl of SYBR Green I Master 2×, 0.3 µl of each 2.5 mM primers with the thermocycling program: 95°C for 10 min, then 95°C for 10 s, 60°C for 30 s, and 72°C for 5 s for 45 cycles plus a dissociation step (60–95°C). The relative expression was calculated as previously described [25]. *Rplp0, Utp6c* and *Eif2a* were selected for normalization as previously described [26].

### Western Blot Analysis

The proteins were isolated from liver that was homogenized in lysis buffer (30 mg of tissue in 1 mL: 20 mM TRIS pH 7.5, 150 mM NaCl, 1% NP-40, 10% glycerol, 5 mM EDTA, 1 mM PMSF and protease inhibitor cocktail Complete Roche) and incubated on an orbital shaker for 1 h at 4°C followed by 15 min centrifugation at 4°C and 12000 rpm. The protein concentration was measured with Pierce BCA Protein Assay kit (Thermo Scientific). The samples were separated by 12% SDS-PAGE and electro-transferred onto a PVDF membrane (Millipore, Eschborn, Germany). The membranes were blocked with 5% (w/v) nonfat dry milk in PBST containing 0.05% or 0.1% (v/v) Tween 20 for 1 h at room temperature. The membranes were then incubated with the appropriate primary antibodies (self-made rabbit polyclonal anti-**CYP51** antibodies against the peptide QRLKDSWAERLDFNPDRY, 1∶2000; anti-**HMGCR**, 1∶1000, ABS229, Millipore, Billerica, MA, USA; anti-**NSDHL**, 1∶250, 15111-1-AP, Proteintech Group, Inc., Chicago, IL, USA; anti-**TM7SF2**, 1∶250, 12033-1-AP, Proteintech Group, Inc., Chicago, IL, USA; anti-**GAPDH** antibodies, 1∶5000, G9545, Sigma Aldrich, St. Louis, MO, USA). Visualization was performed by goat anti-rabbit IgG peroxidase conjugate (1∶5000, A0545, Sigma Aldrich, St. Louis, MO, USA) with 5% nonfat dry milk in PBST and chemiluminescence recorded.

### Statistical analysis

The data was processed and analyzed using different R [27] and Bioconductor [28] packages. Linear regression modeling was employed using three predictor variables corresponding to genotype (*Cyp51^+/−^* and *Cyp51^+/+^*), sex (Female and Male) and diet (LFnC, HFnC and HFC) and 39 response variables corresponding to organ characteristics (7 variables), plasma parameters (7 variables), and hepatic gene expression (25 variables).

The package *car* [29] was used to estimate univariate normalizing transformation of the response variables by Box-Cox power transformation family on the predictors. The Box-Cox transformation of data was necessary, as gene expression results rarely present normal distribution and they were transformed with parameter λ close to 0, which is similar to logarithmic transformation. The transformed response variables were modeled using a linear modeling approach and empirical Bayes smoothing to the standard errors by the *limma* package [30]. A linear model was fitted using three predictor variables and their pairwise interactions.

Benjamini-Hochberg's method for controlling the false discovery rate (FDR) [31] was used to determine statistically significant response variables at significance level of α = 0.1. The significance level of α = 0.1 was chosen, due to small number of animals analyzed in each group. We wished to assess diet/gender/genotype effects on multiple and heterogeneous levels (i.e. globally). As this is (to our best knowledge) the first study of such kind, we have chosen a rather relaxed level of statistical significance (α = 0.1) in order not to miss more subtle, but important effects. The parameters that reached statistical difference close to α = 0.1 will be discussed as the trend and tendencies, but the main focus of the manuscript were differences significant at the level of α = 0.05. Differences between genotypes were assessed across all the mice as well as separately for each diet, sex and their combinations. HFnC and HFC diets were compared to the LFnC diet across all the mice as well as separately for each sex and genotype. Interaction between the diets and genotypes were estimated across all the mice, as well as separately for each sex. Figure S1 presents the hierarchical tree for assessing the *Cyp51* genotype effect. Particular contrasts within the tree allow also studies of the diet – genotype interactions. The numbers of animals analyzed within each contrast (organs characteristics, plasma parameters, liver histology and hepatic gene expression) are shown in Table S2.

## Results

**Figure 2:**
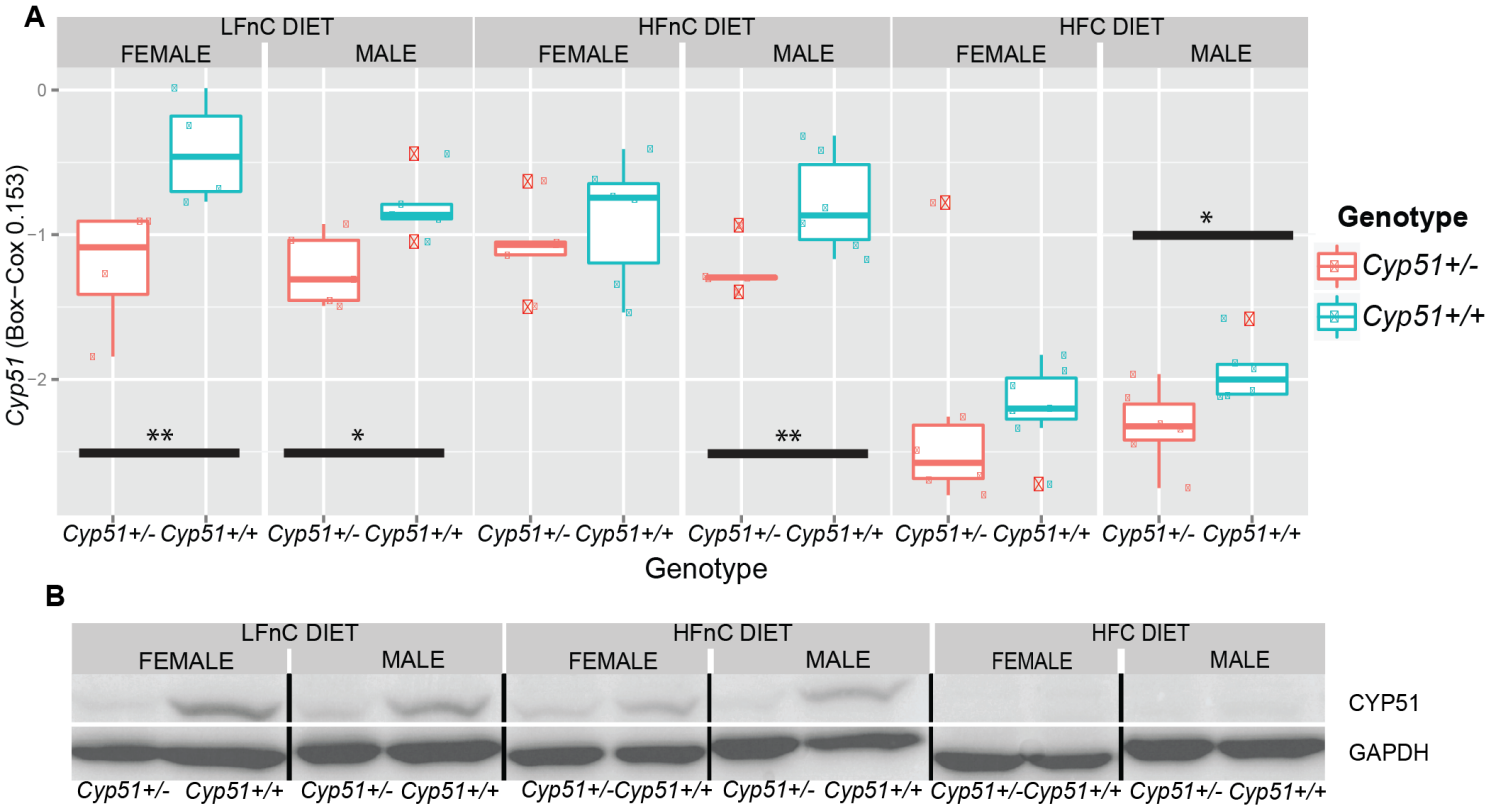
The expression of *Cyp51* (A) on mRNA and (B) protein levels (western blot analysis).

The *Cyp51^+/−^* animals on the standard laboratory diet (LFnC) develop normally based on standard visual inspection and as expected they reproduce normally [18]. The *Cyp51^+/−^* heterozygotes show a decreased expression of *Cyp51* mRNA and protein compared to the *Cyp51^+/+^* controls on each diet (Figure 2), indicating that *Cyp51* is transcribed from both alleles with no compensatory mechanisms in *Cyp51^+/−^* mice. In wild types, the expression is higher on the two diets with no cholesterol (LFnC and HFnC compared to HFC), irrespective of the presence of excess dietary fats (HFnC) and is expectedly repressed on the high-fat, high-cholesterol (HFC) diet due to the cholesterol feedback regulation. A thorough investigation of the blood lipid panel, liver histology, and gene expression by linear regression modeling revealed other significant differences between *Cyp51^+/−^* mice and wild types.

### The global effect of the *Cyp51^+/−^* genotype in males and in females

**Figure 3:**
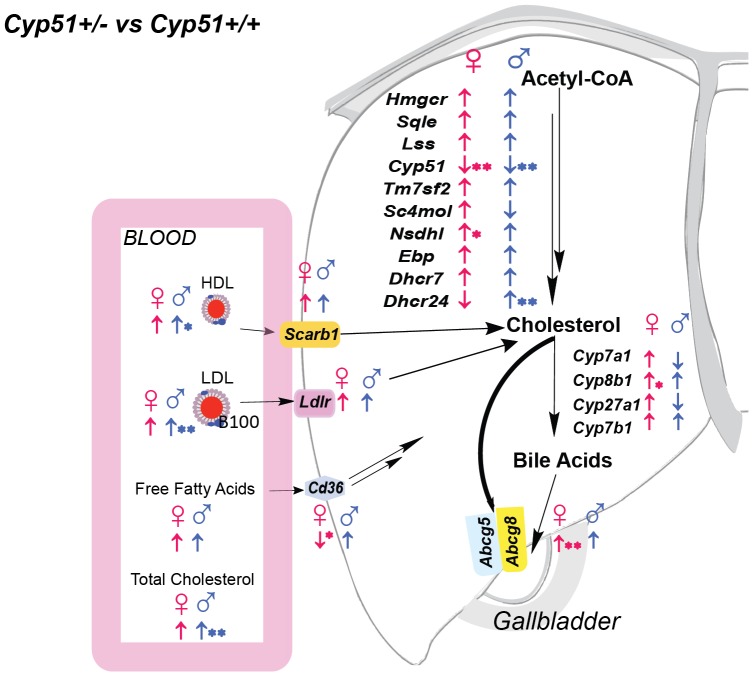
The global effect of *Cyp51* haploinsufficiency. The genotype effect shown on blood lipid profile (n = 8–15) and hepatic gene expression analyses in *Cyp51^+/−^* mice compared to *Cyp51^+/+^* matched with sex (n = 4–7) and blood lipid profile differences. The direction of arrows indicate (↑) up-regulation or (↓) down-regulation of expression in heterozygous females (♀) and males (♂). The direction of arrows shows also a trend of change in genes that did not reach statistical significance. *(p<0.1); **(p<0.05). Results are joined for all diets.

Initially we evaluated the effect of *Cyp51* genotype in sex-matched animals regardless of the feeding regime. This can simulate a random population where feeding is not controlled. The measured values of the body and organ weights, plasma lipids, steroid hormones and relative gene expression are shown in Table S3 with statistically significant features summarized in Table 1 and Figure 3. *Cyp51^+/−^* males had significantly increased plasma total cholesterol, HDL and LDL cholesterol (20–35% higher than controls) and present hepatomegaly (Table 1). On the other hand, *Cyp51^+/−^* females remained leaner than wild-type controls, with the plasma lipid profile similar to that of controls. The hepatic gene expression is summarized in Figure 3. As expected, the *Cyp51^+/−^* mice exhibited a decreased expression of *Cyp51* and a trend of increased expression of majority of other cholesterogenic genes. The observed sexual differences (*Nsdhl* increased in females, *Dhcr24* increased in males) require further investigation. On top of that, *Cyp51^+/−^* females had significantly increased expression of *Abcg8* transporter, responsible for the cholesterol clearance to the bile, as well as the tendency of the increased expression of *Cyp8b1* from alternative bile acid synthesis, and slightly decreased expression of fatty acid translocase *Cd36*. These changes in gene expression could aid in maintaining the healthier blood lipid profile of *Cyp51^+/−^* females compared to the males. The results from pooled population where mice ate either of the diets suggest that *Cyp51* haploinsufficiency is more harmful for males than females.

**Table 1:** Significant differences in gross phenotype between *Cyp51^+/−^* and *Cyp51^+/+^* mice.

	Females *Cyp51^+/−^*		Females *Cyp51^+/+^*			Males *Cyp51^+/−^*		Males *Cyp51^+/+^*		
	Mean	SEM	Mean	SEM	P value	Mean	SEM	Mean	SEM	P value
FAT TISSUE/BW^a^^,^ b	**0.048**	**0.002**	**0.059**	**0.004**	**0.033	0.065	0.004	0.065	0.005	0.984
LIVER/BW^a^^,^ b	4.25	0.058	4.38	0.075	0.326	**4.51**	**0.124**	**4.13**	**0.091**	**0.029
HDL-CHOLESTEROL [mM]	1.43	0.115	1.28	0.076	0.369	**1.98**	**0.103**	**1.66**	**0.091**	* **0.069**
LDL-CHOLESTEROL [mM]	1.09	0.117	0.91	0.102	0.127	**1.52**	**0.168**	**1.13**	**0.135**	**0.029
TOTAL CHOLESTEROL [mM]	2.92	0.234	2.58	0.172	0.194	**3.92**	**0.255**	**3.18**	**0.212**	**0.029

a BW – body weight;

b organ weights/body weight ratios are given in organ weight[g]*100/body weigh [g].

*p<0.1, **p<0.05.

### Liver histology

**Figure 4:**
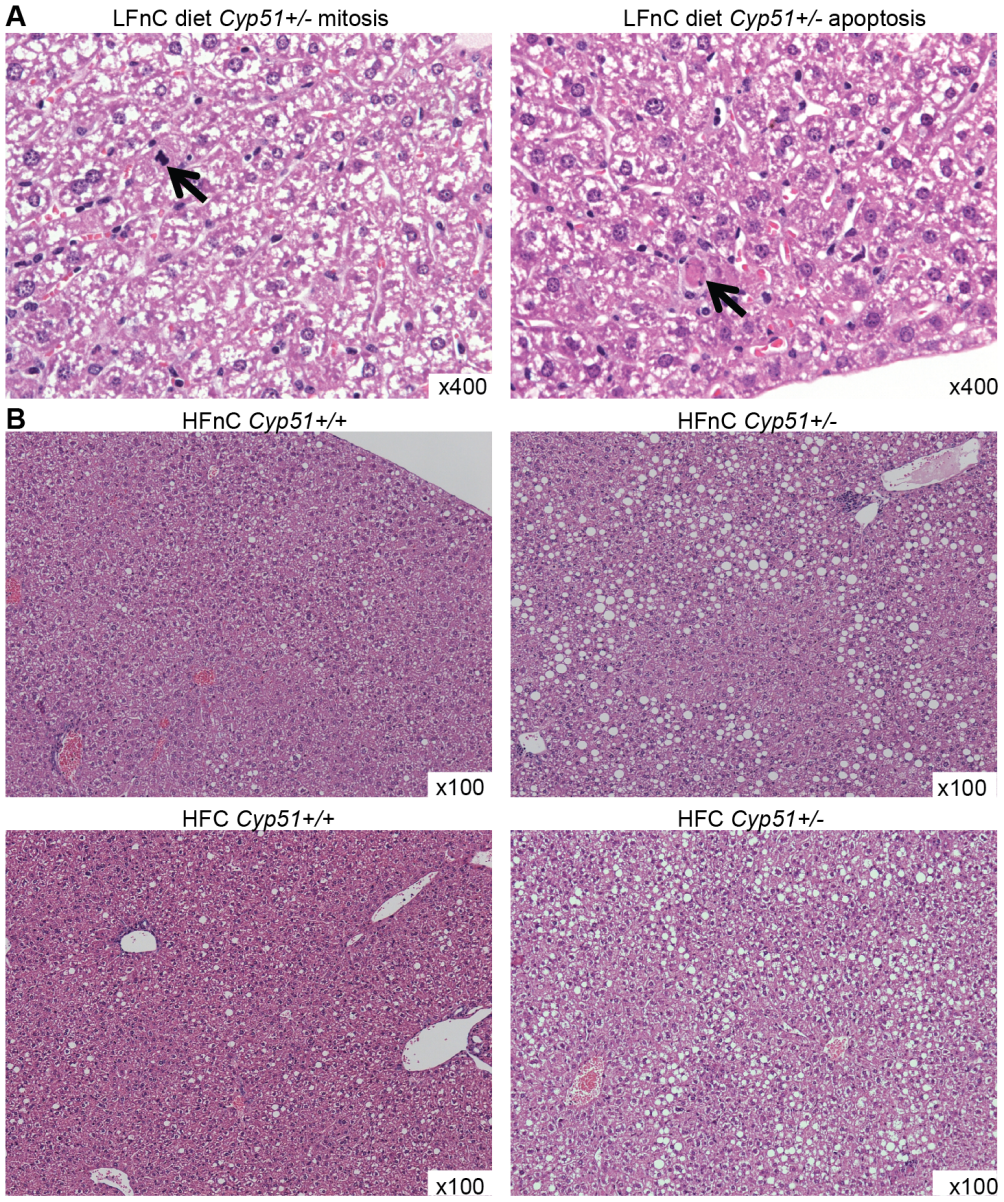
Liver histology, hematoxylin and eosin stain. **A** Mitotic (left) and apoptotic cells with hyper-eosinophilic cytoplasm containing fragments of dense basophilic nuclear material (right) in *Cyp51^+/−^* mice on LFnC diet; original magnification x400. **B** Mild focal steatosis in *Cyp51^+/+^* mice and moderate steatosis in *Cyp51^+/−^* mice on HFnC and HFC diets; original magnification x100.

While the global model suggested hepatomegaly only in *Cyp51^+/−^* males, the histopathology shows similar features in livers of both sexes, thus results are not separated according to the sex (Table 2 and Figure 4). On the standard laboratory diet (LFnC), livers of the *Cyp51^+/−^* mice show frequent apoptoses and mitoses compared to the wild-type mice. The apoptoses and mitoses were still present in *Cyp51^+/−^* mice on the high fat diets with or without cholesterol, yet less frequent than before, suggesting that dietary lipids partially rescued this phenotype. However, both high-fat diets in *Cyp51^+/−^* mice resulted in a moderate steatosis compared to mild steatosis of wild-types, indicating that whole body CYP51 and/or normal cholesterol synthesis may play a role in the hepatic accumulation of the fats.

**Table 2:** Summary of the liver histology.

Histopathology	LFnC^a^		HFnC^b^		HFC^c^	
	*Cyp51^+/−^*	*Cyp51^+/+^*	*Cyp51^+/−^*	*Cyp51^+/+^*	*Cyp51^+/−^*	*Cyp51^+/+^*
Apoptosis, mitosis	Frequent(A = 25; M = 8)^d^	No(A = 4; M = 0)^d^	Rare(A = 7; M = 2)^d^	No(A = 1; M = 0)^d^	Rare(A = 4; M = 1)^d^	No(A = 4; M = 0)^d^^,^ e
Steatosis	No	No	Yes moderate	Yes mild	Yes moderate	Yes mild

a LFnC – low fat without cholesterol diet;

b HFnC – high fat without cholesterol diet;

c HFC – high fat diet with cholesterol;

d A – apoptosis, M – mitosis (number of observed events);

e 5 instead of 6 animals were analyzed.

### Are *Cyp51^+/−^* males at a higher disease risk on high-fat high-cholesterol diet?

In this part, we investigated how mice with one missing *Cyp51* allele react to either a low-fat or high-fat diet, with or without cholesterol. On LFnC standard laboratory diet (low-fat, cholesterol-free), both *Cyp51^+/−^* and wild-types matched by sex had similar body and organ weights, as well as the plasma lipid levels (Table 3). In addition to the significantly reduced *Cyp51* in heterozygotes of both sexes, *Abcg5* transporter, which plays a role in the clearance of cholesterol to the bile, was increased in both sexes and *Dhcr24* only in males (Figure S2 A).

**Table 3:** Significant differences between *Cyp51^+/−^* and *Cyp51^+/+^* mice on each diet separately.

	LFnC Females		LFnC Males		HFnC Females		HFnC Males		HFC Females		HFC Males	
parameter	FC	P.value	FC	P.value	FC	P.value	FC	P.value	FC	P.value	FC	P.value
Body Weight	0.97	0.954	1.04	0.808	1.00	0.993	1.05	0.883	**0.87**	**0.039	1.03	0.712
Fat Tissue/BW^a^^,^ b	1.00	0.954	1.00	0.911	0.84	0.466	0.95	0.893	**0.63**	**0.006	1.09	0.712
Heart/BW^a^^,^ b	0.95	0.954	1.04	0.828	1.07	0.589	0.94	0.835	**1.11**	*0.085	0.95	0.617
Spleen/BW^a^^,^ b	1.03	0.954	1.17	0.808	1.09	0.993	0.94	0.966	1.00	0.578	**0.79**	*0.080
Liver/BW^a^^,^ b	0.95	0.954	1.04	0.808	1.02	0.993	1.01	0.982	0.95	0.351	**1.23**	**0.001
Corticosterone	0.86	0.954	0.77	0.808	0.87	0.993	0.86	0.997	**2.22**	*0.085	0.60	0.617
*Sqle*	0.66	0.134	1.19	0.702	1.38	0.263	1.48	0.132	**2.50**	**0.014	1.00	0.822
*Cyp51*	**0.41**	**0.003	**0.60**	*0.070	0.78	0.435	**0.57**	**0.048	1.57	0.644	**0.60**	*0.090
*Tm7sf2*	0.80	0.151	1.10	0.506	1.29	0.108	1.08	0.662	**1.47**	*0.060	1.13	0.633
*Nsdhl*	0.79	0.288	1.16	0.677	**1.81**	**0.006	1.18	0.410	**1.83**	**0.035	1.11	0.788
*Dhcr7*	0.89	0.507	0.75	0.496	**1.71**	*0.080	1.19	0.566	0.78	0.732	1.11	0.768
*Dhcr24*	0.90	0.573	**1.50**	**0.028	0.95	0.662	**1.29**	*0.098	1.03	0.949	0.93	0.843
*Cyp8b1*	1.00	0.860	1.03	0.908	1.29	0.362	1.06	0.651	**2.00**	**0.011	1.04	0.663
*Cyp27a1*	1.02	0.709	0.85	0.317	1.16	0.381	1.07	0.798	**1.59**	**0.009	1.09	0.767
*Cyp7b1*	1.00	0.566	1.46	0.251	1.67	0.181	1.09	0.935	**3.00**	**0.018	0.97	0.787
*Cd36*	1.32	0.420	1.00	0.894	0.92	0.667	1.00	0.632	**0.68**	*0.063	1.39	0.194
*Abcg5*	**2.25**	*0.073	**2.00**	*0.069	1.67	0.272	0.60	0.159	0.57	0.235	0.74	0.338

FC – fold change calculated as a ratio between average values for *Cyp51^+/−^* mice and average values for *Cyp51^+/+^* mice.

a BW – body weight;

b organ weights/body weight ratios are given in organ weight[g]*100/body weigh [g].

On the diet with no cholesterol but with high fats (HFnC) there was similarly no changes in the body, organ weights and plasma lipids between *Cyp51^+/−^* and wild-type mice of both sexes. Sexual dimorphism was observed at the mRNA level of cholesterol synthesis genes. The majority of measured genes from this pathway were up-regulated in *Cyp51^+/−^* mice even if different genes show statistical significance in males and in females – the overexpression of *Nsdhl* and *Dhcr7* in females and again *Dhcr24* in males (Figure S2 B). A tendency of high NSDHL protein level was observed in both sexes. (Figure S3). From these data, we can deduce that the *Cyp51^+/−^* genotype is not immediately harmful for animals if at the high fat, cholesterol-free diet.

A dramatic influence on *Cyp51^+/−^* mice was observed on the high fat diet with 1.25% of cholesterol (HFC). The *Cyp51^+/−^* females remain significantly leaner compared to wild-types, with lower fat tissue/body weight ratio, and lower body weight, with increased plasma corticosterone (Table 3). The *Cyp51^+/−^* males have significantly enlarged livers compared to the wild-types. The expression of several genes from the cholesterol and bile acid synthesis is significantly increased in *Cyp51^+/−^* females compared to their controls. In females, the mRNA levels of *Tms7Sf2 and Nsdhl* were significantly higher in *Cyp51^+/−^* mice compared to controls (Figure S2 C), and *TMS7SF2* also on the protein level, while the NSDHL protein dropped below the detection threshold (Figure S3 and Figure S4). This can be explained by the overall repression of cholesterol synthesis on the high-fat diet with cholesterol compared to other diets (Table S3). Interestingly, it appears that *Cyp51^+/−^* females repress cholesterol synthesis less effectively than males. From other pathways we noted a decreased expression of fatty acid translocase *Cd36* in *Cyp51^+/−^* females, but not in males (Figure S2 C).

To further investigate the sexual dimorphism, we analyzed the *Cyp51* genotype-sex interactions (Figure S5), which confirmed the significant differences in organ characteristics and in the expression of *Cd36*. The significant differences were found also in the expression of *Cyp27a1* and *Cyp7b1* from the alternative bile acid synthesis pathway, where *Cyp51^+/−^* females exhibited increased expression. *Cyp27a1* is involved in bile acid synthesis pathway, but can also metabolize sterol intermediates from the cholesterol synthesis, including lanosterol [32]. The *Cyp7b1* may also be involved in the development of atherosclerosis [33], oxysterol metabolism, and sex hormone synthesis. The elevated expression of this gene can play a role in the protective effect on cholesterol levels in *Cyp51^+/−^* females. On the other hand, the increase of *Lpl* expression was determined in the *Cyp51^+/−^* males, which should not be the case in the livers of adult animals [34] and can be indicative of an early phases of metabolic syndrome.

The analysis of diet-genotype interactions confirmed observed sexual dimorphisms, resulting in different patterns of gene expression in males and females. The cholesterogenic genes *Nsdhl, Sqle, Cyp51* and *Sc4mol* increase in *Cyp51^+/−^* females when fed with HFnC diet compared to LFnC diet and decrease with cholesterol supplementation (HFC), whereas they are decreased in wild-type females when fed with either of the high-fat diets. On the protein level, we confirmed the decrease in NSDHL expression in *Cyp51^+/−^* females on HFC diet, and the decrease in its expression in wild-type females on both high-fat diets. The expression of *Nsdhl*, *Sqle,* and *Tm7sf2* was still increased in lean *Cyp51^+/−^* females fed HFC compared to their wild-type counterparts, suggesting that the feedback regulation of their expression might be less efficient. The high-fat feeding triggered differential expression of *Abcg5* transporter in *Cyp51^+/−^* males compared to the wild types, whereas in females more diet-genotype interactions were observed. The analysis of interactions did not discover differences in expression of nuclear receptors, leaving the mechanism of the observed phenotype unclear.

### The *Cyp51* genotype interactions with the diet: cholesterol or other dietary fats?

**Figure 5:**
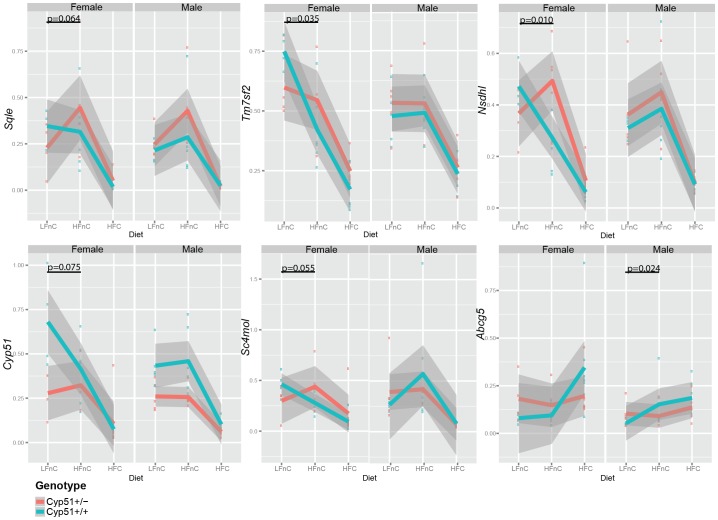
The diet-genotype interactions (effect of high fat feeding). Plots showing the change in expression levels in males and females, both *Cyp51^+/−^* (red) and *Cyp51^+/+^* (blue). Grey color indicates a linear smooth of 0.95 confidence intervals. The significant interactions between factors (*Cyp51* genotype and diet) that can be understood as the differences between slopes of lines are indicated by their p values.

We questioned how the dietary fats affect the *Cyp51^+/−^* genotype is affected, in the absence of cholesterol (HFnC *vs* LFnC) or in the presence of cholesterol (HFC *vs* HFnC), which is the usual case in high-fat nutrition. The major impact was observed for the cholesterol and lipid homeostasis genes, which is shown in the expression plots (Figure 5).

The LFnC and HFnC diets were without significant amounts of cholesterol (<0.01%), so there is a need for the increased cholesterol synthesis. However, females and males adapt to this condition differently. Only females with the *Cyp51^+/−^*genotype increase the expression of genes from cholesterol synthesis on HFnC diet, while the adaptation is already present in wild-type males (Figure 5, Table S4). The difference is observed also at the level of *Abcg5* (from direct clearance of cholesterol), where only the wild-type males, but not females increase the expression on high fat load (comparing LFnC and HFnC) (Figure 5, Table S4).

The cholesterol feedback regulation does not differ majorly between the *Cyp51^+/−^* and wild-type mice in the presence of dietary cholesterol, which is shown in an interaction study between *Cyp51^+/−^ vs Cyp51^+/+^* genotypes and high-fat diets with and without cholesterol (HFnC *vs* HFC). Results indicate that irrespective of the *Cyp51* genotype, mice reacted similarly to cholesterol supplementation in the high-fat milieu (Table S4). Differences in the expression of *Ldlr* in females and *Lpl* in males were significant, but remained in the same direction in both genotypes (Figure S6, Table S4).

## Discussion

The genotype-phenotype correlation is a complex feature, where genes interacting with the environment can lead to less predictable phenotypes even in single-gene knockout models. To assess the global role of the decreased expression of *Cyp51* from cholesterol synthesis, we investigated mice with one functional *Cyp51* allele since the inactivation of both alleles led to the embryonically lethal phenotype [17]. We applied a controlled environment which mimics the human eating habits - mice of both sexes were on diets for 16 weeks and not only 1 to 8 weeks as used in many rodent studies [35], [36], including these from the Mouse Phenome Database (http://phenome.jax.org). A large data-set from the *Cyp51^+/−^* mice and corresponding controls was collected and analyzed by linear regression modeling taking into account the sexual dimorphism, which is characteristic for cholesterol homeostasis in rodents and humans [36], [37], [38].

It appears on first glance, the *Cyp51* heterozygous knockout has a negligible effect, because mice seem to develop normally. A deeper analysis revealed significant changes in the phenotype on the organ and metabolic level. Overall, the *Cyp51^+/−^* males seem to be more affected than *Cyp51^+/−^* females, presenting hepatomegaly and an increased plasma lipid profile. The liver histology showed apoptotic and mitotic events in the *Cyp51^+/−^* mice of both sexes and on all diets (less severe with the high-fat), which was not observed in the wild-type mice and should not be found in livers of healthy animals. The hepatic apoptoses and mitoses are indication of pathological events in cells (such as toxicity, increased oxidative stress, increased accumulation of intermediates, inflammation, etc.), and they are biomarkers for severity of nonalcoholic fatty liver disease (NAFLD) [39]. The *Cyp51^+/−^* females were leaner than wild-type littermates and did not show significant changes in the blood lipid profile compared to wild-types. They also did not show hepatomegaly, but displayed hepatic apoptoses, mitoses and increased lipid infiltration on high-fat diets, which may relate to a group of difficult to diagnose cases of “lean NAFLD” [40]. The extrapolation of metabolic data obtained from murine models directly to humans is not always straightforward. Additionally, the mRNA levels do not necessarily reflect the protein levels and enzymes activity. In our study we found a good correlation between mRNA and protein levels for CYP51 and NSDHL (and partially also for TM7SF2). This is true also for HMGCR where the expression did not differ significantly between the genotypes at the mRNA or protein levels (Figure S7). The liver is a major organ of cholesterol synthesis in mice, but in primates the synthesis mainly occurs in extra hepatic tissues. However, murine models proved to be a great tool investigating inborn errors of cholesterol and bile acid synthesis and phenotypes relate well to human individuals [9], [10]. It is thus plausible to speculate that human individuals of both sexes with functional polymorphisms in the *CYP51A1* are at increased risk to develop liver pathologies.

Our results indicate that the diminished expression of *Cyp51* from cholesterol synthesis was more harmful for males than for females. The male predominance was observed also for *Dhcr24* as another cholesterol synthesis gene, where the viable *Dhcr24^−/−^* knockout males showed a more severe phenotype than females [41]. However, in our case, the female *Cyp51^+/−^* phenotype resembles the ‘lean NAFLD’ with increased corticosterone on the high-fat diet, which might represent another risk factor for rapid development of fatty liver disease. Additionally, the expression of genes from cholesterol synthesis in *Cyp51^+/−^* females appears to be more sensitive to the high-fat diet challenge. It appears that females (even *Cyp51* wild-types) are sensing lack of cholesterol better than males (especially on the high fat diet) increasing the expression of cholesterol synthesis genes more efficiently than males, which improves the flux through the cholesterol synthesis pathway towards cholesterol [36].

The expression of cholesterogenic genes was shown to be sex-biased; however, data reported in literature are inconsistent [36], [42], [43]. We identified higher expression of several genes in females including *Srebp2*, *Hmgcr*, *Lss* and *Ebp* from cholesterol synthesis (Table S5), which is in line with Gatti *et al* [43] and relates well with Lorbek et al [36]. However, in our study we investigated sexual dimorphism in context of *Cyp51* genotype and we uncovered novel differences at the level of expression of cholesterogenic genes. Knockout of one *Cyp51* allele led to a reduced expression of the CYP51 protein in both sexes. The *Cyp51* haploinsufficiency triggered the increased expression of *Dhcr24* in males and *Nsdhl* in females. DHCR24 metabolizes different precursor sterols, including lanosterol which is the major substrate of CYP51 [44]. NSDHL accumulates on the lipid droplets where it is involved in storage of neutral lipids, such as triacylglycerol and cholesterol esters [45] and possibly contributes to the cholesterol clearance in *Cyp51^+/−^* females. The *Nsdhl* gene is located on the X chromosome and possibly due to a presence of a single copy cannot be overexpressed in males, but it seems to play an important role in the cholesterol homeostasis of *Cyp51^+/−^* females. The leaner *Cyp51^+/−^* females showed the increased expression of *Cyp8b1* from alternative pathway of bile acid synthesis and *Abcg8* from transport of cholesterol to bile suggesting increased cholesterol clearance. The decreased adipose tissue correlates with a healthier blood lipid profile in *Cyp51^+/−^* females and decreased expression of *Cd36* accounting for the protective effect from cardiovascular diseases [46], [47] in *Cyp51^+/^*females, but not in males. The mechanism of observed sex differences cannot be explained by our current experimental design, but one possible explanation for sex differences could be in the interaction of lowered *Cyp51* expression with female sex hormones [48], [49]. The sex hormone hypothesis is supported by the study of Romero-Aleshire *et al* [50] demonstrating that in the mouse menopause model, even without a high-fat diet treatment, progression into menopause caused a significant increase in the cholesterol levels indicating that estrogens might indeed play a role.

The global model focusing on differences observed only due to *Cyp51* genotype show that *Cyp51^+/−^* males had significantly increased total cholesterol and LDL cholesterol compared to *Cyp51^+/+^* mice, while *Cyp51^+/−^* females managed to maintain blood lipids on similar level as wild-types, possibly due to the increased clearance *via Abcg5/8* transporters or modified lipids storage. Apparently, the diminished expression of *Cyp51* was still sufficient for cholesterol synthesis, even in the absence of cholesterol in the diet, but resulted in increased mRNA of other cholesterogenic genes and that further led to dysregulation of lipid homeostasis. Heterozygous mice showed increased plasma cholesterol concentration, increased hepatic lipid storage and signs of metabolic stress (indicated by hepatic apoptotic and mitotic events in both sexes and increased corticosterone in females). The mRNA levels of nuclear receptors could not explain this phenotype and the mechanism of dysregulation of lipid homeostasis is yet to be discovered. The decrease in *Cd36* and increase in gene expression from the bile acid synthetic pathway on HFC diet likely contributed to better management of the healthy blood lipid profile in *Cyp51^+/−^* females, but significantly increased corticosterone might represent an independent fatty liver risk factor [51]. Corticosterone is a stress hormone that, when elevated, can lead to changes in metabolism of carbohydrates, proteins, and lipid homeostasis, resulting also in the leaner phenotype in mice fed with high-fat diet [52]. The increased corticosterone does not always affect cholesterol levels in rodents [53], which was also the case in *Cyp51^+/−^* females. Because *Cyp51^+/−^* females were housed in the same manner as wild-types at all diet regimes, the increased corticosterone in *Cyp51^+/−^* HFC group suggests metabolic stress that could be a consequence of the loss of one *Cyp51* allele.

## Conclusions

The heterozygous mutations leading to the decreased protein levels or enzyme activity often result in mild phenotypes that can be easily overlooked. The cumulative effects of single mutations, which may cause very mild phenotypes independently, together with the environmental factors, carry important information about causes of complex diseases. It seems that much of the genetic control of common diseases is due to rare variants with a strong impact on disease risk in individual patients [54]. Our work shows that even heterozygous loss of function of one *Cyp51* allele from the cholesterol synthesis leads to significant phenotype differences in male and female mice, where *Cyp51^+/−^* males are more affected by harmful cholesterol lipid profile and hepatomegaly, but also *Cyp51^+/−^* females reveal risk factors of the fatty liver disease. It is thus plausible to assume that the etiology of liver diseases differs between males and females, where cholesterol synthesis represents a crucial point of sexual dimorphism.

## Supporting Information

Figure S1 Outline of the statistical analysis.
(TIF)

Figure S2 The genotype effect on hepatic gene expression and blood lipids for (A) low fat, cholesterol-free diet; (B) high fat, cholesterol-free diet; and (C) High fat with 1.25% of cholesterol added diet. The direction of arrows indicate (↑) up-regulation or (↓) down-regulation of expression in heterozygous females (♀) and males (♂). * indicates p<0.1 and **p<0.05.
(TIF)

Figure S3 The expression of *Nsdhl* (A) on mRNA and (B) protein levels (western blot analysis) **p<0.05.
(TIF)

Figure S4 The expression of *Tm7sf2* (A) on mRNA and (B) DHCR14 protein levels (western blot analysis) *p<0.1.
(TIF)

Figure S5 The significant interactions between sex and *Cyp51* genotype in mice fed with the high-fat diet with 1.25% of cholesterol - the organ characteristics and the hepatic gene expression. The interactions in organ characteristics/blood profile were not observed on cholesterol-free diets. The *Dhcr24* and *Abcg8* were significantly different (p = 0.06 and 0.07 respectively) on the LFnC diet and *Abcg5* reached significance (p = 0.08) on the HFnC diet (data not shown).
(TIF)

Figure S6 The significant interactions between the *Cyp51* genotype and two high-fat diets with and without cholesterol.
(TIF)

Figure S7 The expression of *Hmgcr* (A) on mRNA and (B) protein levels (western blot analysis) **p<0.05.
(TIF)

Table S1 The list of primers used for RT-PCR analysis.
(DOCX)

Table S2 Number of animals analyzed within each contrast; A – body characteristics B – plasma lipids profile C – hepatic gene expression, D – liver histology.
(DOCX)

Table S3 The average values (Mean) for weights, lipid profile and gene expression and standard error means (SEM) for global model (regardless diets) and for each diet separately.
(DOCX)

Table S4 The interactions between diets and genotypes. Left panel shows the significant interactions between two cholesterol-free diets and *Cyp51* genotype (indicated p values). The directions of arrows indicate the directions of changes from LFnC diet to HFnC diet, stars indicate statistically significant changes (** p<0.05 and * p<0.1). The right panel shows the significant interactions (p values indicated) between two high-fat diets and *Cyp51* genotype. The directions of arrows indicate directions of changes for HFnC to HFC diet and stars indicate statistically significant changes.
(DOCX)

Table S5 The sexual dimorphism – differences between females and males expressed as FC (females mean/males mean). *p<0.1; **p<0.05.
(DOCX)
