# Supplementary figures and images for: Hidden Disease Susceptibility and Sexual Dimorphism in the Heterozygous Knockout of Cyp51 from Cholesterol Synthesis
Source: PLoS One. 2014 Nov 13;9(11):e112787. doi: 10.1371/journal.pone.0112787 (PMC4231084; doi:10.1371/journal.pone.0112787)

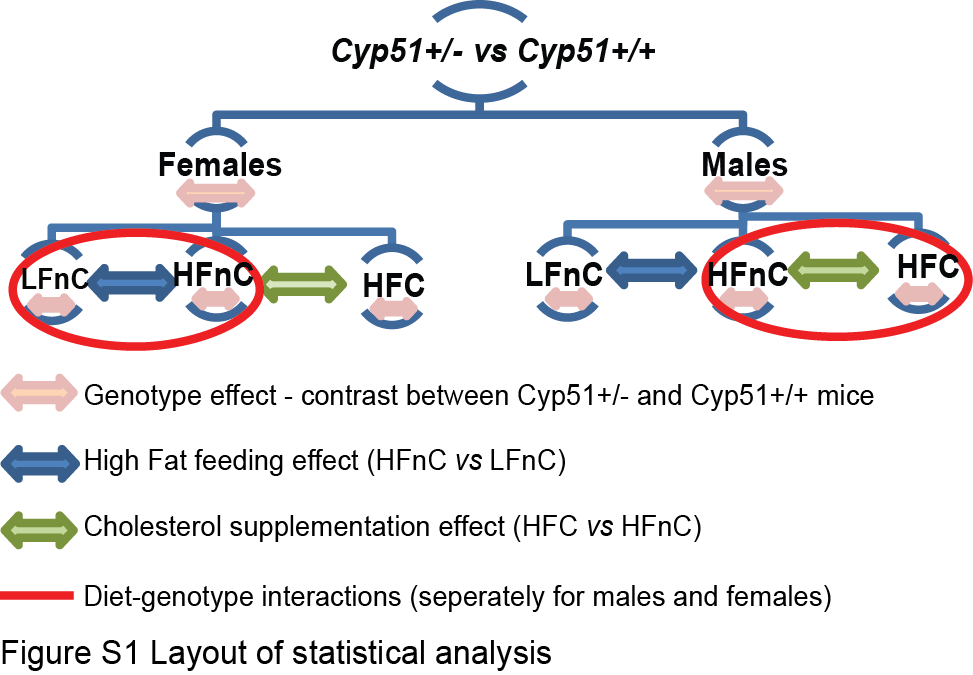

Supplement: Figure S1 — Outline of the statistical analysis. (TIF) [file pone.0112787.s001.tif]

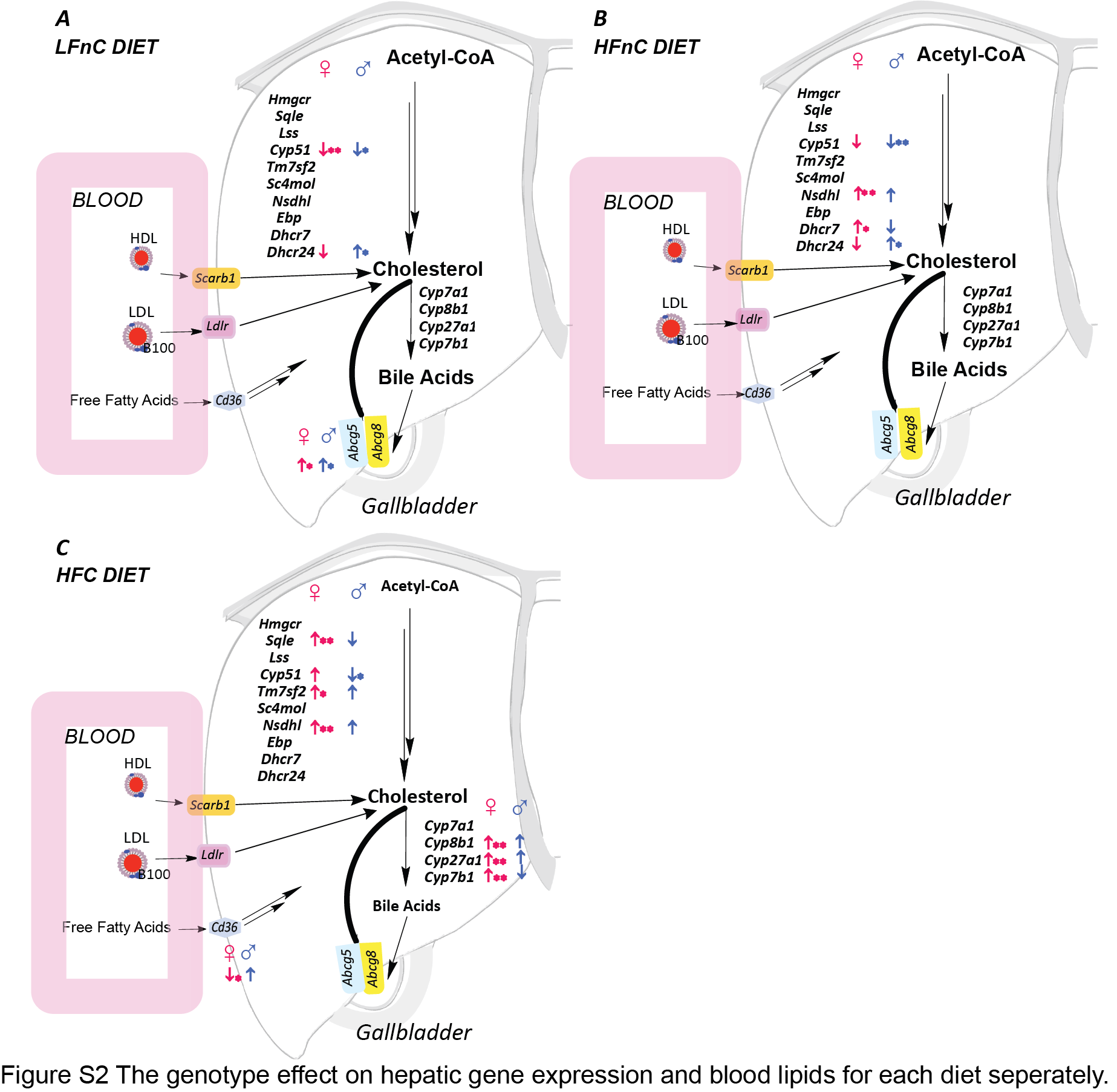

Supplement: Figure S2 — The genotype effect on hepatic gene expression and blood lipids for (A) low fat, cholesterol-free diet; (B) high fat, cholesterol-free diet; and (C) High fat with 1.25% of cholesterol added diet. The direction of arrows indicate (↑) up-regulation or (↓) down-regulation of expression in heterozygous females (♀) and males (♂). * indicates p<0.1 and **p<0.05. (TIF) [file pone.0112787.s002.tif]

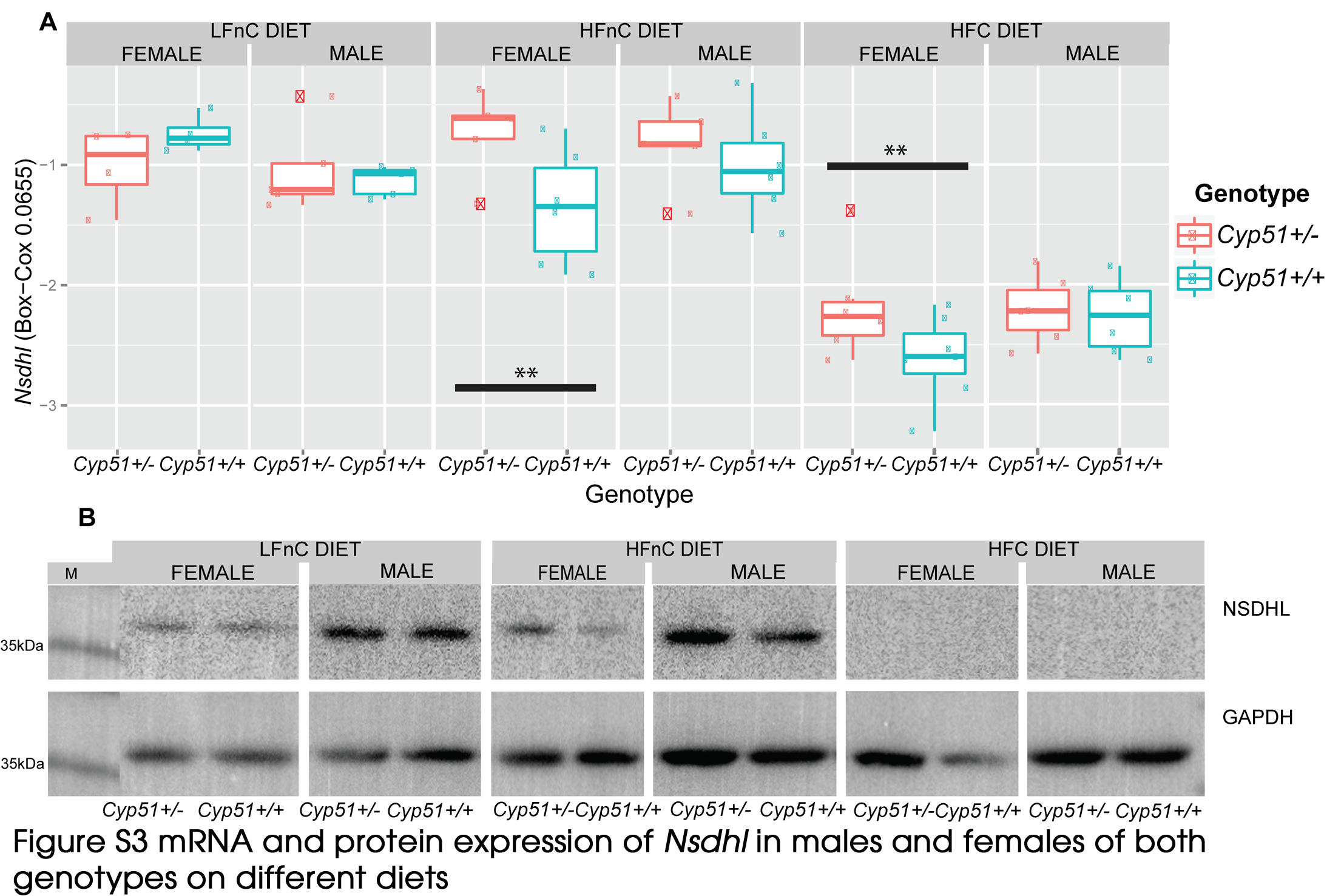

Supplement: Figure S3 — The expression of Nsdhl (A) on mRNA and (B) protein levels (western blot analysis) **p<0.05. (TIF) [file pone.0112787.s003.tif]

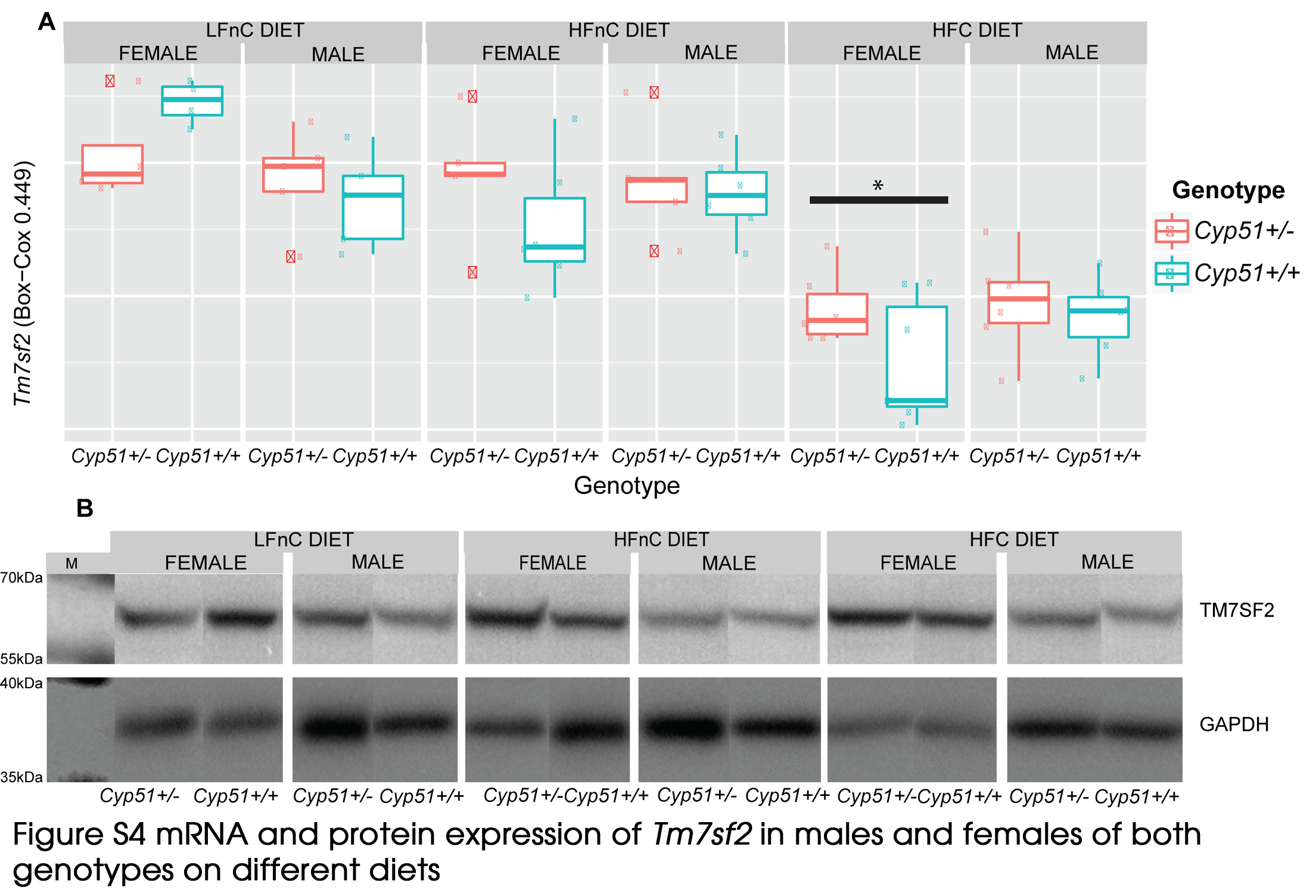

Supplement: Figure S4 — The expression of Tm7sf2 (A) on mRNA and (B) DHCR14 protein levels (western blot analysis) *p<0.1. (TIF) [file pone.0112787.s004.tif]

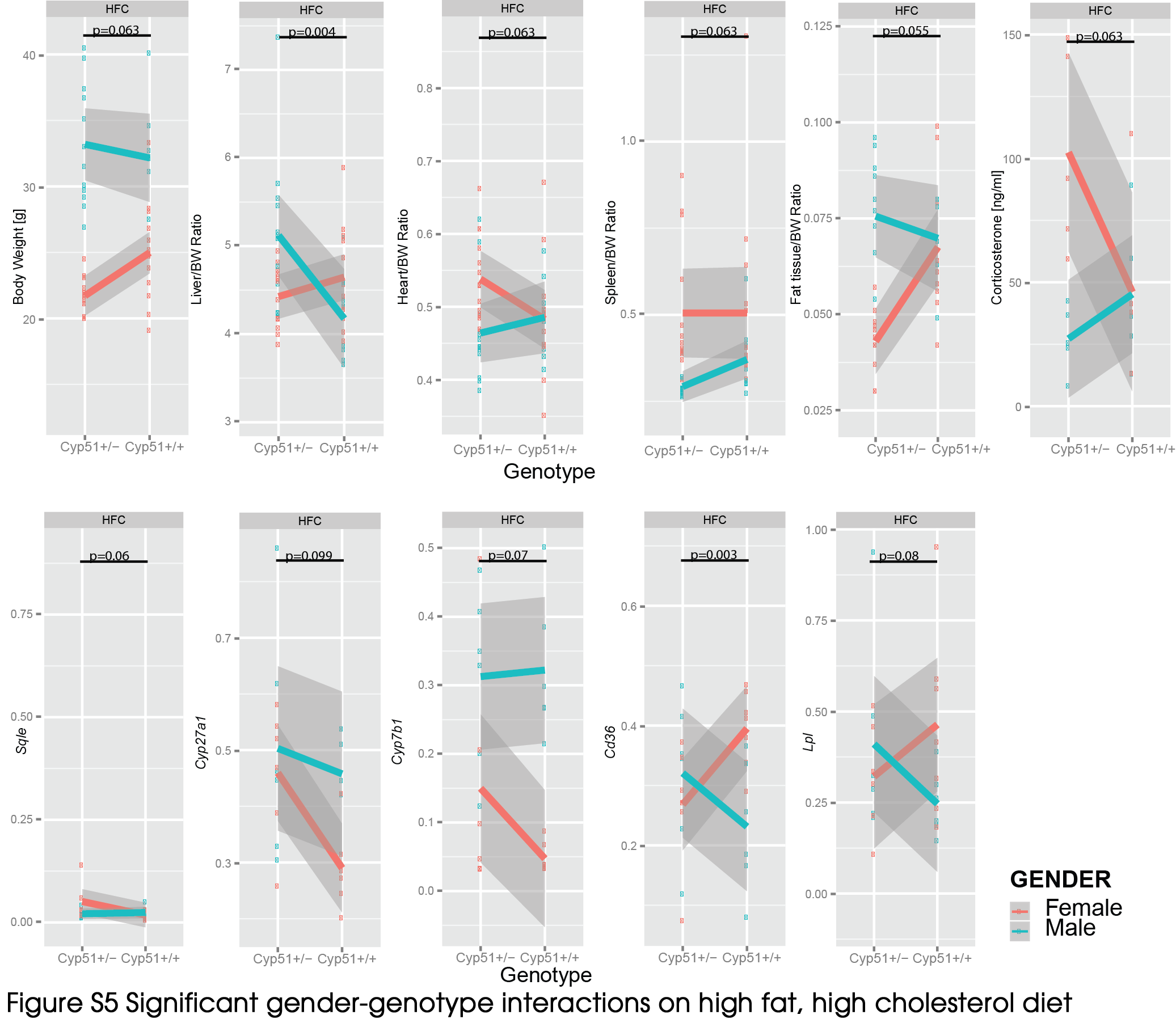

Supplement: Figure S5 — The significant interactions between sex and Cyp51 genotype in mice fed with the high-fat diet with 1.25% of cholesterol - the organ characteristics and the hepatic gene expression. The interactions in organ characteristics/blood profile were not observed on cholesterol-free diets. The Dhcr24 and Abcg8 were significantly different (p = 0.06 and 0.07 respectively) on the LFnC diet and Abcg5 reached significance (p = 0.08) on the HFnC diet (data not shown). (TIF) [file pone.0112787.s005.tif]

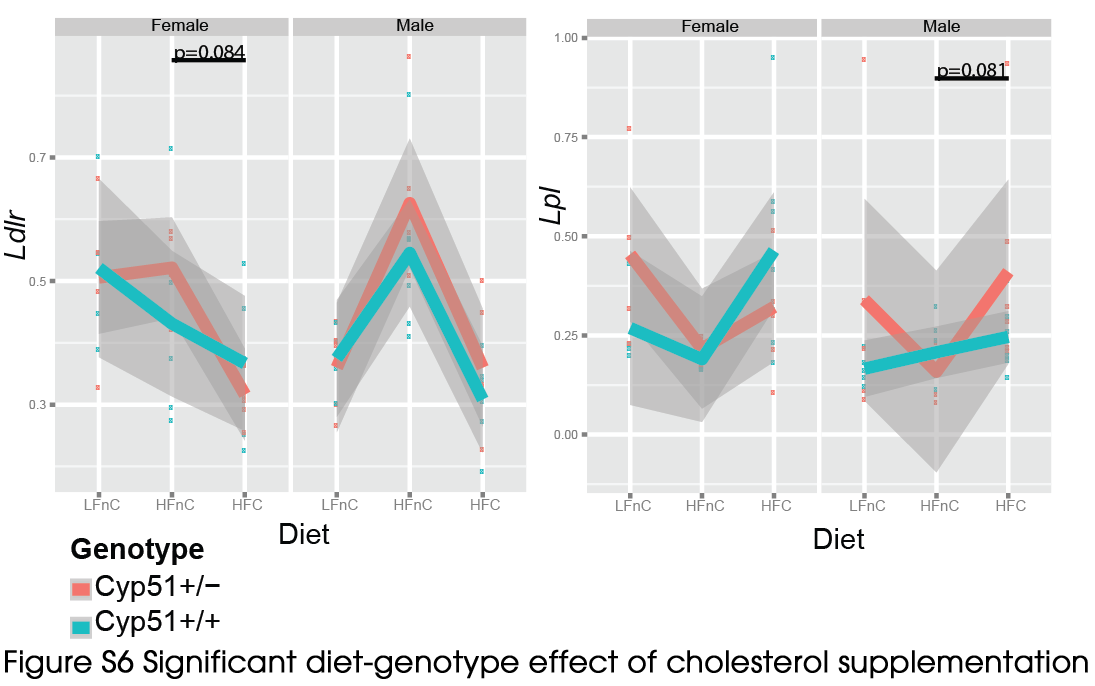

Supplement: Figure S6 — The significant interactions between the Cyp51 genotype and two high-fat diets with and without cholesterol. (TIF) [file pone.0112787.s006.tif]

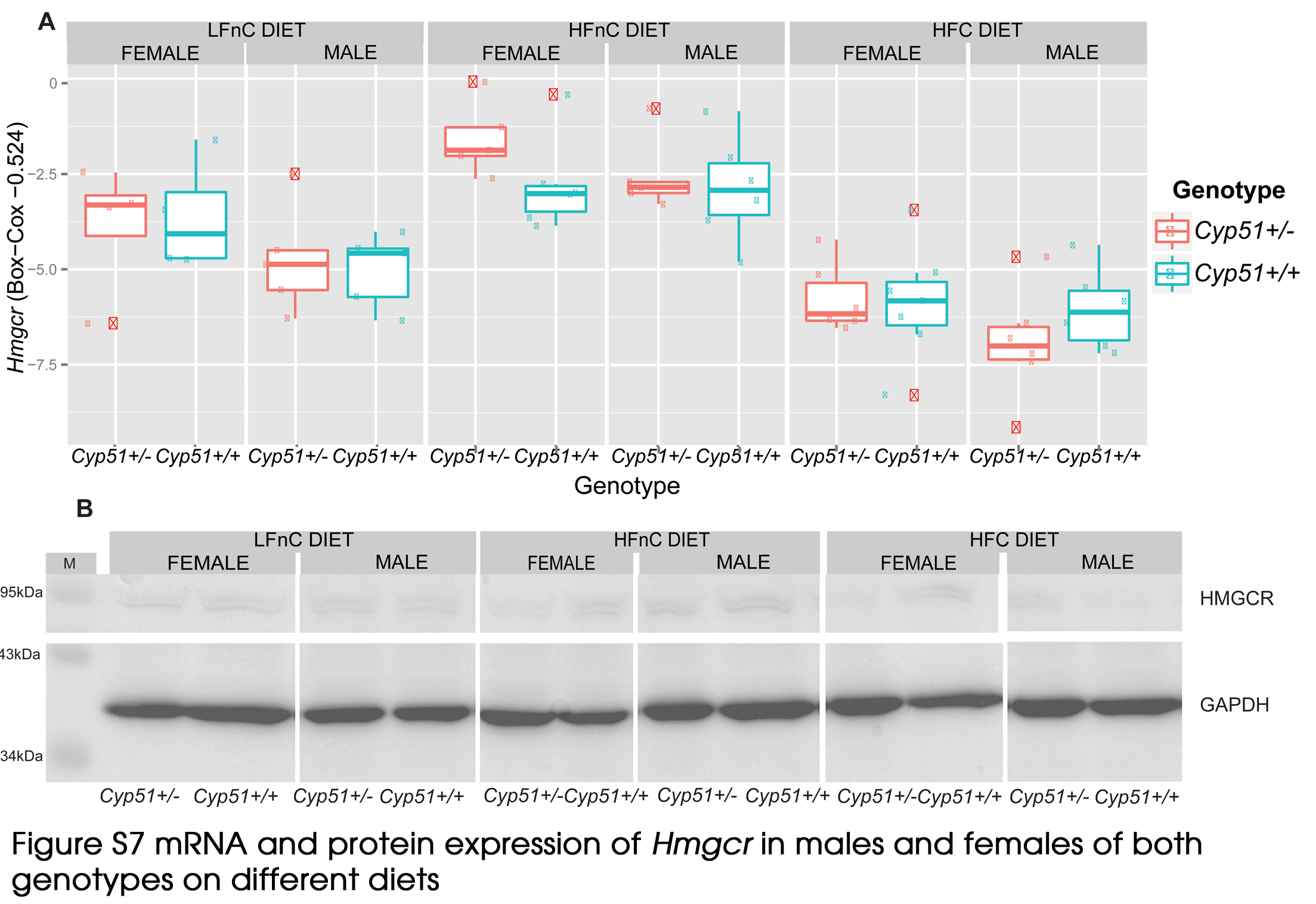

Supplement: Figure S7 — The expression of Hmgcr (A) on mRNA and (B) protein levels (western blot analysis) **p<0.05. (TIF) [file pone.0112787.s007.tif]
