# Supplementary material for: Hidden Disease Susceptibility and Sexual Dimorphism in the Heterozygous Knockout of Cyp51 from Cholesterol Synthesis
Source: PLoS One. 2014 Nov 13;9(11):e112787. doi: 10.1371/journal.pone.0112787 (PMC4231084; doi:10.1371/journal.pone.0112787)
Supplement: Table S1 — The list of primers used for RT-PCR analysis. (DOCX) [file pone.0112787.s008.docx]

**Table S1** List of primers used for RT-PCR

| Gene | Primer | Sequence |
| --- | --- | --- |
| *Rplp0* | fw | CACTGGTCTAGGACCCGAGAAG |
|  | rw | GGTGCCTCTGGAGATTTTCG |
| *Eif2a* | fw | CAACGTGGCAGCCTTACA |
|  | rw | TTTCATGTCATAAAGTTGTAGGTTAGG |
| *Utpc6c* | fw | TTTCGGTTGAGTTTTTCAGGA |
|  | rw | CCCTCAGGTTTACCATCTTGC |
| *Actb* | fw | CCGTGAAAAGATGACCCAGATC |
|  | rw | CACAGCCTGGATGGCTACGT |
| *Gapdh* | fw | CCAATGTGTCCGTCGTGGATCT |
|  | rw | GTTGAAGTCGCAGGAGACAACC |
| *Hprt1* | fw | TCCTCCTCAGACCGCTTTT |
|  | rw | CCTGGTTCATCATCGCTAATC |
| *Hmgcr* | fw | CTTGTGGAATGCCTTGTGATTG |
|  | rw | AGCCGAAGCAGCACATGAT |
| *Sqle* | fw | TCAACCCCAGTCCAGTTCTC |
|  | rw | GACTCCTTCAGGTGCTCAGG |
| *Lss* | fw | GGCACCAATGGATCACAGAT |
|  | rw | AAACTCAGGTCTGTGGTGTGC |
| *Cyp51* | fw | ACGCTGCCTGGCTATTGC |
|  | rw | TTGATCTCTCGATGGGCTCTATC |
| *Tm7sf2* | fw | GCCTCGGTTCCTTTGACTT |
|  | rw | ATCAGCAGGGCCAGGTTA |
| *Sc4mol* | fw | CGGAATTGTGCTTTTGTGTG |
|  | rw | GCGGGTTGAGAGGAATATCA |
| *Nsdhl* | fw | TGCAGCTCTAGGTGGAAAGG |
|  | rw | GAACGTCCAGAAAGGGATTG |
| *Ebp* | fw | TGTCCTACAGCTTGTGGTGTCT |
|  | rw | AATAAACGGGGTGGCCTATC |
| *Dhcr7* | fw | GCTTCAGGCAGGCACTTAGA |
|  | rw | GGATTTCGAAGCCATCAGG |
| *Dhcr24* | fw | GGTCATGACGGACGACGTA |
|  | rw | AGGGCTTGTAGTAACTGCCAAT |
| *Ldlr* | fw | AGGCTGTGGGCTCCATAGG |
|  | rw | TGCGGTCCAGGGTCATCT |
| *Scarb1* | fw | TCAGAAGCTGTTCTTGGTCTGAAC |
|  | rw | GTTCATGGGGATCCCAGTGA |
| *Cd36* | fw | AAAACGACTGCAGGTCAACA |
|  | rw | CATTTCTGCTTTTTCATCACCA |
| *Abcg5* | fw | CTTACCCACGGTTCCTTTCA |
|  | rw | ACGCATAATCACTGCCTGCT |
| *Abcg8* | fw | AAGACGGGCTGTACACTGCT |
|  | rw | AGTAGATGGGCATCGCGTAG |
| *Lpl* | fw | GGACTGAGAATGGCAAGCAA |
|  | rw | CCACTGTGCCGTACAGAGAAA |
| *Srebp-2* | fw | GCGTTCTGGAGACCATGGA |
|  | rw | ACAAAGTTGCTCTGAAAACAAATCA |
| *Pparg* | fw | GGTTGACACAGAGATGCCATTCT |
|  | rw | AATGCGAGTGGTCTTCCATCA |
| *Lxr* | fw | ACTTCAGTTACAACCGGGAAGA |
|  | rw | GCTCTGGAGAACTCAAAGATGG |
| *Car* | fw | CAGGGTTCCAGTACGAGTTTTG |
|  | rw | AGGCTCCTGGAGATGCAGTC |
| *Cyp7a1* | fw | CAGGGAGATGCTCTGTGTTCA |
|  | rw | AGGCATACATCCCTTCCGTGA |
| *Cyp7b1* | fw | CCGATTCTGCCGTCTCCTT |
|  | rw | GCAGCCTTACTCTGCAAAGCTT |
| *Cyp8b1* | fw | AAGGCTGGCTTCCTGAGCTT |
|  | rw | AACAGCTCATCGGCCTCATC |
| *Cyp27a1* | fw | CCTCACCTATGGGATCTTCATC |
|  | rw | TTTAAGGCATCCGTGTAGAGC |
