# Supplementary material for: Hidden Disease Susceptibility and Sexual Dimorphism in the Heterozygous Knockout of Cyp51 from Cholesterol Synthesis
Source: PLoS One. 2014 Nov 13;9(11):e112787. doi: 10.1371/journal.pone.0112787 (PMC4231084; doi:10.1371/journal.pone.0112787)
Supplement: Table S2 — Number of animals analyzed within each contrast; A – body characteristics B – plasma lipids profile C – hepatic gene expression, D – liver histology. (DOCX) [file pone.0112787.s009.docx]

|  | | Female | | Male | |
| --- | --- | --- | --- | --- | --- |
| Diet |  | *Cyp51^+/-^* | *Cyp51^+/+^* | *Cyp51^+/-^* | *Cyp51^+/+^* |
| LFnC | A | 15 | 12 | 13 | 8 |
|  | B | 15 | 12 | 13 | 8 |
|  | C | 4 | 4 | 5 | 5 |
|  | D | 3 | 3 | 3 | 3 |
| HFnC | A | 15 | 12 | 12 | 8 |
|  | B | 12 | 10 | 12 | 8 |
|  | C | 5 | 6 | 5 | 6 |
|  | D | 3 | 2 | 3 | 3 |
| HFC | A | 14 | 13 | 12 | 8 |
|  | B | 13 | 13 | 11 | 8 |
|  | C | 6 | 7 | 6 | 6 |
|  | D | 3 | 3 | 3 | 3 |

**Table S2** - Number of animals analyzed within each contrasts
A – body characteristics B – plasma lipids profile C – hepatic gene expression, D – liver histology
