# Supplementary material for: Hidden Disease Susceptibility and Sexual Dimorphism in the Heterozygous Knockout of Cyp51 from Cholesterol Synthesis
Source: PLoS One. 2014 Nov 13;9(11):e112787. doi: 10.1371/journal.pone.0112787 (PMC4231084; doi:10.1371/journal.pone.0112787)
Supplement: Table S3 — The average values (Mean) for weights, lipid profile and gene expression and standard error means (SEM) for global model (regardless diets) and for each diet separately. (DOCX) [file pone.0112787.s010.docx]

**Table S3.** The average values (Mean) for weights, lipid profile and gene expression and standard error means (SEM) for global model (regardless diets) and for each diet separately.

|  | Diet | Global effect (all diets) | | | | LFnC | | | | HFnC | | | | HFC | | | |
| --- | --- | --- | --- | --- | --- | --- | --- | --- | --- | --- | --- | --- | --- | --- | --- | --- | --- |
|  | Sex | Female | | Male | | Female | | Male | | Female | | Male | | Female | | Male | |
|  | *Cyp51* genotype | +/- | +/+ | +/- | +/+ | +/- | +/+ | +/- | +/+ | +/- | +/+ | +/- | +/+ | +/- | +/+ | +/- | +/+ |
| Body weight [g] | Mean | 21.88 | 23.25 | 31.62 | 30.54 | 21.11 | 21.87 | 28.25 | 27.24 | 22.79 | 22.75 | 33.72 | 32.23 | 21.72 | 24.98 | 33.19 | 32.16 |
|  | SEM | 0.368 | 0.525 | 0.747 | 0.917 | 0.31 | 0.54 | 0.5 | 0.96 | 0.96 | 0.85 | 1.35 | 1.63 | 0.34 | 1.04 | 1.32 | 1.54 |
| Brain weight [g] | Mean | 0.439 | 0.451 | 0.426 | 0.432 | 0.429 | 0.454 | 0.435 | 0.439 | 0.441 | 0.447 | 0.417 | 0.44 | 0.449 | 0.453 | 0.424 | 0.417 |
|  | SEM | 0.003 | 0.003 | 0.005 | 0.005 | 0.006 | 0.004 | 0.008 | 0.008 | 0.006 | 0.005 | 0.01 | 0.008 | 0.005 | 0.007 | 0.008 | 0.009 |
| Fat tissue/Bw | Mean | 0.048 | 0.059 | 0.065 | 0.065 | 0.046 | 0.046 | 0.047 | 0.047 | 0.054 | 0.064 | 0.07 | 0.074 | 0.043 | 0.068 | 0.076 | 0.07 |
|  | SEM | 0.002 | 0.004 | 0.004 | 0.005 | 0.002 | 0.002 | 0.003 | 0.006 | 0.005 | 0.008 | 0.008 | 0.008 | 0.002 | 0.007 | 0.006 | 0.005 |
| Heart/bw | Mean | 0.524 | 0.503 | 0.494 | 0.501 | 0.523 | 0.55 | 0.572 | 0.55 | 0.511 | 0.479 | 0.439 | 0.466 | 0.538 | 0.483 | 0.464 | 0.486 |
|  | SEM | 0.01 | 0.013 | 0.016 | 0.014 | 0.013 | 0.015 | 0.027 | 0.029 | 0.02 | 0.023 | 0.021 | 0.017 | 0.016 | 0.022 | 0.021 | 0.02 |
| Kidneys/bw | Mean | 0.97 | 0.937 | 0.973 | 0.96 | 1.008 | 1.013 | 1.055 | 1.076 | 0.955 | 0.913 | 0.936 | 0.872 | 0.945 | 0.89 | 0.921 | 0.931 |
|  | SEM | 0.013 | 0.016 | 0.021 | 0.026 | 0.021 | 0.01 | 0.022 | 0.046 | 0.026 | 0.026 | 0.042 | 0.036 | 0.014 | 0.027 | 0.03 | 0.017 |
| Spleen/bw | Mean | 0.451 | 0.437 | 0.317 | 0.331 | 0.406 | 0.393 | 0.35 | 0.298 | 0.444 | 0.407 | 0.305 | 0.326 | 0.505 | 0.505 | 0.293 | 0.371 |
|  | SEM | 0.025 | 0.028 | 0.016 | 0.018 | 0.018 | 0.014 | 0.042 | 0.012 | 0.051 | 0.022 | 0.016 | 0.036 | 0.05 | 0.075 | 0.01 | 0.038 |
| Liver/bw | Mean | 4.248 | 4.382 | 4.505 | 4.129 | 4.204 | 4.423 | 4.341 | 4.185 | 4.125 | 4.054 | 4.068 | 4.026 | 4.427 | 4.647 | 5.121 | 4.176 |
|  | SEM | 0.058 | 0.075 | 0.124 | 0.091 | 0.099 | 0.061 | 0.115 | 0.088 | 0.102 | 0.087 | 0.138 | 0.199 | 0.091 | 0.154 | 0.255 | 0.178 |
| HDL [mM] | Mean | 1.403 | 1.284 | 1.977 | 1.658 | 1.033 | 1.038 | 1.515 | 1.241 | 1.328 | 1.178 | 1.978 | 1.824 | 1.898 | 1.593 | 2.521 | 1.908 |
|  | SEM | 0.115 | 0.076 | 0.103 | 0.091 | 0.041 | 0.042 | 0.119 | 0.108 | 0.141 | 0.088 | 0.066 | 0.128 | 0.282 | 0.156 | 0.206 | 0.13 |
| LDL [mM] | Mean | 1.089 | 0.909 | 1.516 | 1.125 | 0.478 | 0.434 | 0.617 | 0.445 | 1.288 | 0.891 | 1.981 | 1.735 | 1.61 | 1.36 | 2.07 | 1.194 |
|  | SEM | 0.117 | 0.102 | 0.168 | 0.135 | 0.042 | 0.031 | 0.059 | 0.043 | 0.144 | 0.134 | 0.141 | 0.227 | 0.235 | 0.186 | 0.383 | 0.086 |
| Total cholesterol [mM] | Mean | 2.924 | 2.581 | 3.918 | 3.183 | 1.957 | 1.905 | 2.596 | 2.098 | 3.058 | 2.477 | 4.374 | 3.979 | 3.915 | 3.285 | 4.984 | 3.471 |
|  | SEM | 0.234 | 0.172 | 0.255 | 0.212 | 0.066 | 0.083 | 0.18 | 0.161 | 0.27 | 0.168 | 0.159 | 0.324 | 0.555 | 0.355 | 0.568 | 0.207 |

| FFA [mM] | Mean | 1.3 | 1.21 | 1.305 | 1.167 | 1.274 | 1.357 | 1.398 | 1.355 | 1.257 | 1.261 | 1.109 | 1.098 | 1.368 | 1.036 | 1.41 | 1.049 |
| --- | --- | --- | --- | --- | --- | --- | --- | --- | --- | --- | --- | --- | --- | --- | --- | --- | --- |
|  | SEM | 0.071 | 0.065 | 0.06 | 0.082 | 0.073 | 0.105 | 0.102 | 0.203 | 0.082 | 0.104 | 0.09 | 0.067 | 0.193 | 0.11 | 0.104 | 0.117 |
| Triglycerides [mM] | Mean | 0.945 | 0.86 | 0.938 | 0.872 | 0.979 | 0.958 | 1.024 | 0.896 | 0.962 | 0.898 | 0.911 | 0.909 | 0.89 | 0.741 | 0.865 | 0.811 |
|  | SEM | 0.046 | 0.04 | 0.029 | 0.038 | 0.043 | 0.049 | 0.043 | 0.09 | 0.085 | 0.056 | 0.038 | 0.042 | 0.113 | 0.08 | 0.064 | 0.06 |
| Corticosterone [ng/ml] | Mean | 75.16 | 62.8 | 32.67 | 44.18 | 71.23 | 82.63 | 37.34 | 48.32 | 51.79 | 59.7 | 33.46 | 39.02 | 102.45 | 46.07 | 27.22 | 45.19 |
|  | SEM | 10.21 | 10.21 | 7.23 | 7.81 | 19.61 | 19.63 | 21.17 | 14.88 | 7.53 | 16.13 | 7.21 | 15.17 | 18.08 | 16.69 | 5.95 | 13.32 |
| Testosterone [pg/ml] | Mean |  |  | 3.33 | 5.16 |  |  | 5.58 | 4 |  |  | 0.58 | 0.23 |  |  | 3.84 | 11.02 |
|  | SEM |  |  | 1.43 | 2.48 |  |  | 3.7 | 3.36 |  |  | 0.23 | 0.24 |  |  | 2.11 | 6.06 |
| *Hmgcr* | Mean | 0.201 | 0.154 | 0.129 | 0.127 | 0.14 | 0.16 | 0.11 | 0.09 | 0.4 | 0.24 | 0.24 | 0.22 | 0.07 | 0.08 | 0.06 | 0.07 |
|  | SEM | 0.057 | 0.037 | 0.029 | 0.026 | 0.03 | 0.05 | 0.03 | 0.01 | 0.13 | 0.09 | 0.07 | 0.06 | 0.01 | 0.01 | 0.01 | 0.01 |
| *Sqle* | Mean | 0.23 | 0.211 | 0.219 | 0.182 | 0.23 | 0.35 | 0.25 | 0.21 | 0.44 | 0.32 | 0.43 | 0.29 | 0.05 | 0.02 | 0.02 | 0.02 |
|  | SEM | 0.062 | 0.05 | 0.052 | 0.044 | 0.07 | 0.05 | 0.04 | 0.02 | 0.13 | 0.08 | 0.09 | 0.09 | 0.02 | 0 | 0 | 0.01 |
| *Lss* | Mean | 0.276 | 0.226 | 0.199 | 0.177 | 0.21 | 0.23 | 0.2 | 0.17 | 0.51 | 0.34 | 0.34 | 0.27 | 0.12 | 0.13 | 0.08 | 0.09 |
|  | SEM | 0.061 | 0.038 | 0.037 | 0.027 | 0.05 | 0.03 | 0.05 | 0.02 | 0.13 | 0.08 | 0.07 | 0.05 | 0.02 | 0.03 | 0.01 | 0.01 |
| *Cyp51* | Mean | 0.224 | 0.337 | 0.184 | 0.325 | 0.28 | 0.68 | 0.26 | 0.43 | 0.32 | 0.41 | 0.26 | 0.46 | 0.11 | 0.07 | 0.06 | 0.1 |
|  | SEM | 0.043 | 0.071 | 0.028 | 0.051 | 0.06 | 0.13 | 0.04 | 0.05 | 0.05 | 0.07 | 0.03 | 0.08 | 0.07 | 0.01 | 0.01 | 0.01 |
| *Tm7sf2* | Mean | 0.44 | 0.396 | 0.429 | 0.396 | 0.6 | 0.75 | 0.53 | 0.48 | 0.54 | 0.42 | 0.53 | 0.49 | 0.25 | 0.17 | 0.26 | 0.23 |
|  | SEM | 0.052 | 0.062 | 0.044 | 0.037 | 0.07 | 0.03 | 0.06 | 0.05 | 0.07 | 0.06 | 0.07 | 0.04 | 0.03 | 0.03 | 0.04 | 0.03 |
| *Sc4mol* | Mean | 0.295 | 0.246 | 0.281 | 0.308 | 0.3 | 0.46 | 0.39 | 0.27 | 0.44 | 0.28 | 0.42 | 0.57 | 0.17 | 0.09 | 0.07 | 0.08 |
|  | SEM | 0.058 | 0.043 | 0.059 | 0.093 | 0.08 | 0.06 | 0.14 | 0.02 | 0.1 | 0.04 | 0.05 | 0.23 | 0.09 | 0.03 | 0.01 | 0.01 |
| *Nsdhl* | Mean | 0.305 | 0.232 | 0.29 | 0.259 | 0.37 | 0.47 | 0.36 | 0.31 | 0.49 | 0.27 | 0.45 | 0.38 | 0.11 | 0.06 | 0.1 | 0.09 |
|  | SEM | 0.053 | 0.046 | 0.049 | 0.041 | 0.06 | 0.04 | 0.07 | 0.02 | 0.07 | 0.06 | 0.07 | 0.08 | 0.03 | 0.01 | 0.01 | 0.01 |
| *Ebp* | Mean | 0.56 | 0.503 | 0.487 | 0.437 | 0.65 | 0.66 | 0.51 | 0.46 | 0.65 | 0.54 | 0.54 | 0.5 | 0.42 | 0.38 | 0.42 | 0.35 |
|  | SEM | 0.047 | 0.042 | 0.031 | 0.023 | 0.1 | 0.08 | 0.06 | 0.02 | 0.07 | 0.05 | 0.02 | 0.03 | 0.05 | 0.05 | 0.06 | 0.03 |

| *Dhcr7* | Mean | 0.356 | 0.303 | 0.29 | 0.284 | 0.31 | 0.35 | 0.24 | 0.32 | 0.53 | 0.31 | 0.44 | 0.37 | 0.21 | 0.27 | 0.2 | 0.18 |
| --- | --- | --- | --- | --- | --- | --- | --- | --- | --- | --- | --- | --- | --- | --- | --- | --- | --- |
|  | SEM | 0.063 | 0.035 | 0.038 | 0.04 | 0.11 | 0.06 | 0.05 | 0.09 | 0.12 | 0.04 | 0.06 | 0.06 | 0.04 | 0.08 | 0.03 | 0.03 |
| *Dhcr24* | Mean | 0.363 | 0.375 | 0.401 | 0.329 | 0.44 | 0.49 | 0.42 | 0.28 | 0.37 | 0.39 | 0.53 | 0.41 | 0.31 | 0.3 | 0.27 | 0.29 |
|  | SEM | 0.029 | 0.024 | 0.041 | 0.032 | 0.04 | 0.03 | 0.03 | 0.05 | 0.06 | 0.03 | 0.1 | 0.07 | 0.04 | 0.02 | 0.01 | 0.04 |
| *Cyp7a1* | Mean | 0.42 | 0.356 | 0.289 | 0.262 | 0.44 | 0.4 | 0.17 | 0.11 | 0.42 | 0.29 | 0.33 | 0.27 | 0.41 | 0.38 | 0.36 | 0.37 |
|  | SEM | 0.046 | 0.045 | 0.035 | 0.034 | 0.14 | 0.11 | 0.02 | 0.02 | 0.05 | 0.06 | 0.05 | 0.05 | 0.07 | 0.08 | 0.06 | 0.04 |
| *Cyp8b1* | Mean | 0.268 | 0.194 | 0.353 | 0.337 | 0.37 | 0.37 | 0.41 | 0.4 | 0.22 | 0.17 | 0.37 | 0.35 | 0.24 | 0.12 | 0.29 | 0.28 |
|  | SEM | 0.039 | 0.029 | 0.024 | 0.03 | 0.09 | 0.07 | 0.04 | 0.04 | 0.04 | 0.02 | 0.03 | 0.05 | 0.06 | 0.01 | 0.04 | 0.05 |
| *Cyp27a1* | Mean | 0.425 | 0.336 | 0.486 | 0.483 | 0.46 | 0.45 | 0.46 | 0.54 | 0.36 | 0.31 | 0.49 | 0.46 | 0.46 | 0.29 | 0.5 | 0.46 |
|  | SEM | 0.039 | 0.023 | 0.04 | 0.027 | 0.13 | 0.02 | 0.07 | 0.07 | 0.02 | 0.04 | 0.06 | 0.04 | 0.05 | 0.03 | 0.08 | 0.04 |
| *Cyp7b1* | Mean | 0.088 | 0.043 | 0.306 | 0.263 | 0.05 | 0.05 | 0.35 | 0.24 | 0.05 | 0.03 | 0.25 | 0.23 | 0.15 | 0.05 | 0.31 | 0.32 |
|  | SEM | 0.031 | 0.004 | 0.032 | 0.024 | 0.01 | 0 | 0.06 | 0.05 | 0.01 | 0 | 0.05 | 0.02 | 0.07 | 0.01 | 0.05 | 0.04 |
| *Ldlr* | Mean | 0.435 | 0.426 | 0.443 | 0.412 | 0.51 | 0.52 | 0.36 | 0.37 | 0.52 | 0.43 | 0.63 | 0.55 | 0.32 | 0.37 | 0.36 | 0.31 |
|  | SEM | 0.033 | 0.034 | 0.041 | 0.034 | 0.07 | 0.07 | 0.03 | 0.02 | 0.03 | 0.07 | 0.06 | 0.06 | 0.02 | 0.04 | 0.04 | 0.03 |
| *Scrab1* | Mean | 0.39 | 0.308 | 0.334 | 0.298 | 0.43 | 0.33 | 0.41 | 0.26 | 0.37 | 0.24 | 0.28 | 0.37 | 0.38 | 0.36 | 0.32 | 0.25 |
|  | SEM | 0.056 | 0.027 | 0.052 | 0.028 | 0.08 | 0.05 | 0.17 | 0.02 | 0.15 | 0.02 | 0.02 | 0.07 | 0.07 | 0.05 | 0.04 | 0.02 |
| *Cd36* | Mean | 0.324 | 0.364 | 0.246 | 0.213 | 0.41 | 0.31 | 0.15 | 0.15 | 0.33 | 0.36 | 0.25 | 0.25 | 0.27 | 0.4 | 0.32 | 0.23 |
|  | SEM | 0.035 | 0.02 | 0.032 | 0.036 | 0.1 | 0.01 | 0.04 | 0.02 | 0.04 | 0.05 | 0.05 | 0.09 | 0.04 | 0.02 | 0.05 | 0.04 |
| *Abcg5* | Mean | 0.176 | 0.194 | 0.112 | 0.135 | 0.18 | 0.08 | 0.1 | 0.05 | 0.15 | 0.09 | 0.09 | 0.15 | 0.2 | 0.35 | 0.14 | 0.19 |
|  | SEM | 0.029 | 0.05 | 0.016 | 0.025 | 0.06 | 0.01 | 0.03 | 0.01 | 0.04 | 0.02 | 0.03 | 0.05 | 0.05 | 0.1 | 0.03 | 0.04 |
| *Abcg8* | Mean | 0.351 | 0.265 | 0.252 | 0.246 | 0.41 | 0.19 | 0.15 | 0.19 | 0.23 | 0.18 | 0.23 | 0.23 | 0.41 | 0.38 | 0.36 | 0.31 |
|  | SEM | 0.058 | 0.032 | 0.033 | 0.023 | 0.2 | 0.02 | 0.02 | 0.02 | 0.02 | 0.03 | 0.03 | 0.03 | 0.06 | 0.04 | 0.06 | 0.04 |
| *Srebp2* | Mean | 0.259 | 0.267 | 0.213 | 0.2 | 0.32 | 0.32 | 0.37 | 0.17 | 0.33 | 0.26 | 0.22 | 0.32 | 0.16 | 0.25 | 0.08 | 0.11 |
|  | SEM | 0.05 | 0.047 | 0.053 | 0.038 | 0.09 | 0.06 | 0.14 | 0.01 | 0.11 | 0.04 | 0.05 | 0.09 | 0.05 | 0.11 | 0.01 | 0.02 |

| *Pxr* | Mean | 0.844 | 0.721 | 0.73 | 0.702 | 1.01 | 0.84 | 0.8 | 0.78 | 0.76 | 0.66 | 0.74 | 0.69 | 0.81 | 0.7 | 0.66 | 0.65 |
| --- | --- | --- | --- | --- | --- | --- | --- | --- | --- | --- | --- | --- | --- | --- | --- | --- | --- |
|  | SEM | 0.095 | 0.056 | 0.06 | 0.054 | 0.35 | 0.18 | 0.06 | 0.1 | 0.03 | 0.06 | 0.11 | 0.07 | 0.08 | 0.08 | 0.13 | 0.12 |
| *Car* | Mean | 0.288 | 0.287 | 0.223 | 0.264 | 0.35 | 0.27 | 0.19 | 0.16 | 0.31 | 0.26 | 0.26 | 0.36 | 0.22 | 0.32 | 0.22 | 0.26 |
|  | SEM | 0.034 | 0.046 | 0.031 | 0.041 | 0.08 | 0.09 | 0.06 | 0 | 0.07 | 0.02 | 0.07 | 0.09 | 0.02 | 0.1 | 0.04 | 0.05 |
| *Lxr* | Mean | 0.343 | 0.334 | 0.342 | 0.342 | 0.38 | 0.35 | 0.29 | 0.31 | 0.33 | 0.34 | 0.38 | 0.37 | 0.33 | 0.32 | 0.36 | 0.34 |
|  | SEM | 0.032 | 0.011 | 0.019 | 0.017 | 0.12 | 0.03 | 0.01 | 0.01 | 0.02 | 0.01 | 0.03 | 0.03 | 0.04 | 0.02 | 0.04 | 0.04 |
| *Pparg* | Mean | 0.332 | 0.292 | 0.373 | 0.352 | 0.513 | 0.255 | 0.161 | 0.226 | 0.243 | 0.261 | 0.415 | 0.392 | 0.314 | 0.334 | 0.415 | 0.421 |
|  | SEM | 0.062 | 0.038 | 0.054 | 0.049 | 0.305 | 0.072 | 0.003 | 0.061 | 0.053 | 0.05 | 0.11 | 0.052 | 0.05 | 0.073 | 0.067 | 0.103 |
| *Lpl* | Mean | 0.322 | 0.321 | 0.31 | 0.209 | 0.454 | 0.269 | 0.34 | 0.166 | 0.217 | 0.19 | 0.159 | 0.208 | 0.322 | 0.464 | 0.41 | 0.246 |
|  | SEM | 0.044 | 0.051 | 0.067 | 0.019 | 0.12 | 0.054 | 0.158 | 0.017 | 0.012 | 0.01 | 0.03 | 0.032 | 0.062 | 0.1 | 0.113 | 0.036 |
