# Supplementary material for: Hidden Disease Susceptibility and Sexual Dimorphism in the Heterozygous Knockout of Cyp51 from Cholesterol Synthesis
Source: PLoS One. 2014 Nov 13;9(11):e112787. doi: 10.1371/journal.pone.0112787 (PMC4231084; doi:10.1371/journal.pone.0112787)
Supplement: Table S4 — The interactions between diets and genotypes. Left panel shows the significant interactions between two cholesterol-free diets and Cyp51 genotype (indicated p values). The directions of arrows indicate the directions of changes from LFnC diet to HFnC diet, stars indicate statistically significant changes (** p<0.05 and * p<0.1). The right panel shows the significant interactions (p values indicated) between two high-fat diets and Cyp51 genotype. The directions of arrows indicate directions of changes for HFnC to HFC diet and stars indicate statistically significant changes. (DOCX) [file pone.0112787.s011.docx]

**Table S4.** The interactions between diets and genotypes.

|  | HFnC vs LFnC & in Females | | | HFnC vs LFnC & in Males | | | HFC vs HFnC & in Females | | | HFC vs HFnC & in Males | | |
| --- | --- | --- | --- | --- | --- | --- | --- | --- | --- | --- | --- | --- |
| gene | +/- | +/+ | *Cyp51^+/-^vs Cyp51^+/+^*  P value | +/- | +/+ | *Cyp51^+/-^vs Cyp51^+/+^*  P value | +/- | +/+ | *Cyp51^+/-^vs Cyp51^+/+^* P value | +/- | +/+ | *Cyp51^+/-^vs Cyp51^+/+^* P value |
| *Sqle* | **↑**** | ↓ | ***0.064** | ↑ | ↑ | 0.439 | **↓**** | **↓**** | 0.349 | **↓**** | **↓**** | 0.220 |
| *Cyp51* | ↑ | **↓*** | ***0.075** | = | = | 0.949 | **↓**** | **↓**** | 0.793 | **↓**** | **↓**** | 0.784 |
| *Tm7sf2* | ↓ | **↓↓**** | ****0.035** | = | = | 0.858 | **↓**** | **↓**** | 0.924 | **↓**** | **↓**** | 0.990 |
| *Sc4mol* | ↑ | ↓ | ***0.055** | ↑ | ↑ | 0.719 | **↓**** | **↓**** | 0.911 | **↓**** | **↓**** | 0.760 |
| *Nsdhl* | ↑ | **↓**** | ****0.010** | ↑ | ↑ | 0.787 | **↓**** | **↓**** | 0.533 | **↓**** | **↓**** | 0.680 |
| *Abcg5* | ↓ | ↑ | 0.509 | ↓ | ↑ | ****0.024** | ↑ | **↑**** | 0.109 | ↑ | ↑ | 0.715 |
| *Ldlr* | ↑ | ↓ | 0.237 | **↑**** | **↑**** | 0.364 | **↓**** | ↓ | ***0.084** | **↓**** | **↓**** | 0.993 |
| *Lpl* | **↓*** | ↓ | 0.535 | ↓ | ↑ | 0.149 | ↑ | **↑**** | 0.246 | **↑↑**** | ↑ | ***0.081** |

Left panel shows the significant interactions between two cholesterol-free diets and *Cyp51* genotype (indicated p values). The directions of arrows indicate the directions of changes from LFnC diet to HFnC diet, stars indicate statistically significant changes (** p<0.05 and * p<0.1). The right panel shows the significant interactions (p values indicated) between two high-fat diets and *Cyp51* genotype. The directions of arrows indicate directions of changes for HFnC to HFC diet and stars indicate statistically significant changes.
