# Supplementary material for: Hidden Disease Susceptibility and Sexual Dimorphism in the Heterozygous Knockout of Cyp51 from Cholesterol Synthesis
Source: PLoS One. 2014 Nov 13;9(11):e112787. doi: 10.1371/journal.pone.0112787 (PMC4231084; doi:10.1371/journal.pone.0112787)
Supplement: Table S5 — The sexual dimorphism – differences between females and males expressed as FC (females mean/males mean). *p<0.1; **p<0.05. (DOCX) [file pone.0112787.s012.docx]

|  |  |  | LFnC | | | | HFnC | | | | HFC | | | |
| --- | --- | --- | --- | --- | --- | --- | --- | --- | --- | --- | --- | --- | --- | --- |
|  | Females vs Males | | *Cyp51^+/-^* | | *Cyp51^+/+^* | | *Cyp51^+/-^* | | *Cyp51^+/+^* | | *Cyp51^+/-^* | | *Cyp51^+/+^* | |
|  | FC | pvalue | FC | pvalue | FC | pvalue | FC | pvalue | FC | pvalue | FC | pvalue | FC | pvalue |
| *Hmgcr* | **1.35** | **0.018**** | 1.28 | 0.312 | 1.16 | 0.103 | 1.84 | 0.241 | 1.00 | 0.893 | 1.39 | 0.112 | 1.11 | 0.814 |
| *Sqle* | 1.07 | 0.413 | 0.90 | 0.490 | 1.46 | 0.209 | 1.01 | 0.954 | 0.97 | 0.726 | **1.28** | **0.050*** | 0.75 | 0.458 |
| *Lss* | **1.30** | **0.044**** | 1.11 | 0.833 | 1.20 | 0.339 | 1.25 | 0.286 | 1.25 | 0.603 | 1.40 | 0.111 | 1.51 | 0.465 |
| *Cyp51* | 1.11 | 0.651 | 1.11 | 0.959 | 1.28 | 0.130 | 1.26 | 0.485 | 0.92 | 0.597 | 0.68 | 0.848 | 0.75 | 0.264 |
| *Tm7sf2* | 1.00 | 0.548 | 1.17 | 0.471 | **1.64** | **0.006**** | 0.94 | 0.873 | 0.84 | 0.286 | 0.81 | 0.862 | 0.73 | 0.115 |
| *Sc4mol* | 1.02 | 0.870 | 1.29 | 0.401 | 1.53 | 0.191 | 0.85 | 0.933 | 0.71 | 0.206 | 1.01 | 0.134 | 1.24 | 0.778 |
| *Nsdhl* | 0.88 | 0.782 | 0.86 | 0.898 | 1.58 | 0.104 | 0.92 | 0.683 | 0.62 | 0.103 | 0.77 | 0.909 | **0.66** | **0.083*** |
| *Ebp* | **1.11** | **0.013**** | 1.31 | 0.137 | **1.36** | **0.032**** | 1.19 | 0.201 | 1.06 | 0.619 | 0.89 | 0.948 | 1.05 | 0.675 |
| *Dhcr7* | 1.09 | 0.396 | 1.67 | 0.623 | 1.33 | 0.591 | 0.81 | 0.573 | 0.85 | 0.607 | 0.92 | 0.851 | 1.55 | 0.401 |
| *Dhcr24* | 1.00 | 0.461 | 1.09 | 0.766 | **1.81** | **0.004**** | **0.61** | **0.031**** | 0.95 | 0.844 | 0.97 | 0.631 | 1.03 | 0.819 |
| *Cyp7a1* | **1.34** | **0.001**** | **3.34** | **0.013**** | **3.52** | **<0.001**** | 1.28 | 0.358 | 0.97 | 0.896 | 1.15 | 0.641 | 1.03 | 0.886 |
| *Cyp8b1* | **0.60** | **<0.001**** | 1.14 | 0.458 | 1.02 | 0.654 | **0.43** | **0.018**** | **0.42** | **0.003**** | 0.57 | 0.217 | **0.39** | **0.001**** |
| *Cyp27a1* | **0.74** | **0.002**** | 0.98 | 0.771 | 0.82 | 0.400 | 0.81 | 0.128 | **0.67** | **0.023**** | 0.82 | 0.724 | **0.62** | **0.007**** |
| *Cyp7b1* | **0.16** | **<0.001**** | **0.16** | **<0.001**** | **0.21** | **<0.001**** | **0.21** | **<0.001**** | **0.12** | **<0.001**** | **0.17** | **<0.001**** | **0.13** | **<0.001**** |
| *Ldlr* | 0.98 | 0.491 | **1.52** | **0.052*** | **1.23** | **0.062*** | 0.88 | 0.266 | **0.73** | **0.068*** | 0.85 | 0.461 | 1.21 | 0.250 |
| *Scrab1* | 1.10 | 0.355 | 1.79 | 0.137 | 1.16 | 0.366 | 0.92 | 0.789 | **0.78** | **0.021**** | 0.93 | 0.578 | **1.42** | **0.077*** |
| *Cd36* | **1.44** | **<0.001**** | **2.96** | **0.002**** | **2.03** | **0.025**** | 1.45 | 0.313 | **1.13** | **0.043**** | 0.97 | 0.464 | **1.61** | **0.016**** |
| *Abcg5* | **1.62** | **0.021**** | 3.60 | 0.143 | 1.32 | 0.196 | 1.27 | 0.155 | 0.59 | 0.284 | 1.65 | 0.258 | 2.27 | 0.172 |
| *Abcg8* | 1.22 | 0.180 | **3.34** | **0.007**** | 1.11 | 0.854 | 1.13 | 0.851 | 0.71 | 0.167 | 1.22 | 0.615 | 1.37 | 0.446 |
| *Srebp2* | **1.43** | **0.006**** | 1.60 | 0.945 | 1.63 | 0.113 | 1.16 | 0.400 | 0.85 | 0.932 | **1.29** | **0.021**** | **2.45** | **0.024**** |
| *Pxr* | 1.14 | 0.282 | 1.86 | 0.931 | 0.94 | 0.873 | 1.13 | 0.752 | 1.06 | 0.886 | 1.09 | 0.152 | 1.18 | 0.360 |
| *Car* | **1.19** | **0.078*** | **2.62** | **0.033**** | 1.19 | 0.219 | 1.34 | 0.515 | 0.75 | 0.556 | 0.99 | 0.687 | 1.29 | 0.851 |
| *Lxr* | 0.97 | 0.852 | 1.66 | 0.520 | 1.20 | 0.439 | 0.94 | 0.449 | 0.83 | 0.662 | 0.83 | 0.529 | 0.92 | 0.805 |
| *Pprg* | 0.82 | 0.751 | **3.19** | **0.072*** | 1.13 | 0.702 | 0.58 | 0.244 | 0.67 | 0.158 | 0.68 | 0.424 | 0.77 | 0.495 |
| *Lpl* | 1.27 | 0.014 | **1.97** | **0.058*** | 1.74 | 0.141 | 1.55 | 0.130 | 0.85 | 0.963 | 0.80 | 0.463 | 1.82 | 0.070 |

Table S5 The sexual dimorphism – differences between females and males expressed as FC (females mean/males mean). *p<0.1; **p<0.05
